# Coffee decoction enhances tamoxifen proapoptotic activity on MCF-7 cells

**DOI:** 10.1038/s41598-020-76445-z

**Published:** 2020-11-11

**Authors:** Megumi Funakoshi-Tago, Kenji Tago, Chin Li, Shingo Hokimoto, Hiroomi Tamura

**Affiliations:** 1 Division of Hygienic Chemistry, Faculty of Pharmacy, Keio University, 1-5-30 Shibakoen, Minato-ku, Tokyo, 105-8512 Japan; 2 Division of Structural Biochemistry, Department of Biochemistry, Jichi Medical University, 3311-1 Yakushiji, Shimotsuke, Tochigi 329-0498 Japan

**Keywords:** Nutritional supplements, Disease prevention, Apoptosis, Breast cancer

## Abstract

The consumption of coffee has been suggested to effectively enhance the therapeutic effects of tamoxifen against breast cancer; however, the underlying molecular mechanisms remain unclear. We herein attempted to clarify how coffee decoction exerts anti-cancer effects in cooperation with tamoxifen using the estrogen receptor α (ERα)-positive breast cancer cell line, MCF-7. The results obtained showed that coffee decoction down-regulated the expression of ERα, which was attributed to caffeine inhibiting its transcription. Coffee decoction cooperated with tamoxifen to induce cell-cycle arrest and apoptotic cell death, which may have been mediated by decreases in cyclin D1 expression and the activation of p53 tumor suppressor. The inclusion of caffeine in coffee decoction was essential, but not sufficient, to induce cell-cycle arrest and apoptotic cell death, suggesting the requirement of unknown compound(s) in coffee decoction to decrease cyclin D1 expression and activate apoptotic signaling cascades including p53. The activation of p53 through the cooperative effects of these unidentified component(s), caffeine, and tamoxifen appeared to be due to the suppression of the ERK and Akt pathways. Although the mechanisms by which the suppression of these pathways induces p53-mediated apoptotic cell death remain unclear, the combination of decaffeinated coffee, caffeine, and tamoxifen also caused cell-cycle arrest and apoptotic cell death, suggesting that unknown compound(s) present in decaffeinated coffee cooperate with caffeine and tamoxifen.

## Introduction

Breast cancer is one of the most prevalent cancers in women worldwide and at least 70% of breast cancers are classified as estrogen receptor α (ERα)-positive^1,2^. Once stimulated with estrogen, ERα translocates from the cytosol to the nucleus, in which it functions as an activated transcription factor. Following a stimulation with estrogen, nuclear-translocated ERα rapidly binds to estrogen response elements (EREs), which alter the expression of target genes, such as c-Myc, trefoil factor 1 (TFF1), Cathepsin D (CTSD), Growth Regulation by Estrogen in Breast cancer 1 (GREB1), and Progesterone Receptor (PgR)^3–5^. The proto-oncogene products, c-myc, GREB1, and TFF1 have been implicated in the cellular proliferation, metastasis, and migration of breast cancer cells^6,7^. The aspartic protease CTSD, a marker of a poor prognosis in breast cancer, is overproduced and hypersecreted by human breast cancer cells^8^. Another target gene of ERα, PgR is also involved in the acceleration of cellular proliferation via the up-regulation of cyclin D1 expression in breast cancer cells^9,10^.

Among drug strategies against breast cancer, ERα is a potential candidate as a therapeutic target in ERα-positive breast cancer. Tamoxifen, an agent that antagonizes estrogen, is one of the commonly used drugs in the treatment of breast cancers because of its inhibitory effects on the transcriptional activity of Erα^11^. Adjuvant therapy using tamoxifen was previously shown to be effective against early-stage ERα-positive breast cancer and prolonged overall survival^12^. However, some breast cancer patients acquire resistance to tamoxifen during the long-term treatment with tamoxifen^13^.

The molecular mechanisms by which breast cancer cells become resistant to tamoxifen have not yet been clarified. Lack of ER expression through the transcriptional repression of the ER gene is the primary mechanism of de novo resistance to tamoxifen in some patients^14^. Mutations in the ER gene and abnormal splicing also confer loss of ER function^15^. These loss of ERα expression and its function involve a switch from initially ERα-positive to ERα-negative tumors. It was also reported that the appearance of activating mutations in ER gene encoding constitutively active ERα acquired endocrine resistance in breast cancer therapy^16,17^. Furthermore, HER2, a receptor tyrosine kinase, is frequently expressed in breast cancer and has been associated with the aggressive phenotype of breast cancer^18–20^. The Ras/MEK/ERK and PI3K/Akt pathways function as downstream signals of HER2, and play critical roles in cell proliferation and survival^21,22^. ERK and Akt phosphorylate Ser-118 or Ser-167 in ERα, and this phosphorylation induces the estrogen-independent transcriptional activation of ERα^23,24^ which may contribute to the development of tamoxifen resistance. The Ras/MEK/ERK signaling cascade and PI3K/AKT pathway are also required for cell proliferation and survival and these signaling pathways were shown to be frequently activated in breast cancers^25,26^.

Coffee is one of the most widely consumed beverages in the world, and coffee decoction contains numerous components that exhibit biological activities. Coffee includes caffeine as the main ingredient, a plant alkaloid, trigonelline, polyphenols, such as chlorogenic acid, caffeic acids, and pyrocatechol, and diterpenes, including cafestol and kahweol^27–30^. Previous epidemiological studies reported that the consumption of coffee reduced the incidence of breast cancer^31^. Furthermore, Rosendahl et al. found smaller invasive primary tumors and a lower percentage of ERα-positive tumors in patients with a moderate (2–4 cups/day) to high (5 cups/day) coffee intake with than in those with low consumption (1 cup/day). Moderate to high coffee consumption has been associated with a lower risk of breast cancer events in tamoxifen-treated patients with ERα-positive tumors^32^. These findings suggested that coffee intake increased curative efficiency by tamoxifen in ERα-positive breast cancer patients. However, the molecular mechanisms by which coffee ingredients reinforce the anti-tumor activity of tamoxifen in ERα-positive breast cancer patients have not yet been elucidated in detail.

In the present study, we attempted to demonstrate that coffee intake enhances the efficiency of breast cancer treatments by examining the effects of coffee decoction on tamoxifen-induced apoptosis in the ERα-positive breast cancer cell line, MCF-7. Furthermore, we evaluated the effect of caffeine and other components in coffee decoction on tamoxifen-induced apoptosis**.**

## Results

### The co-treatment with coffee and tamoxifen induces cell-cycle arrest and apoptosis

**Figure 1:**
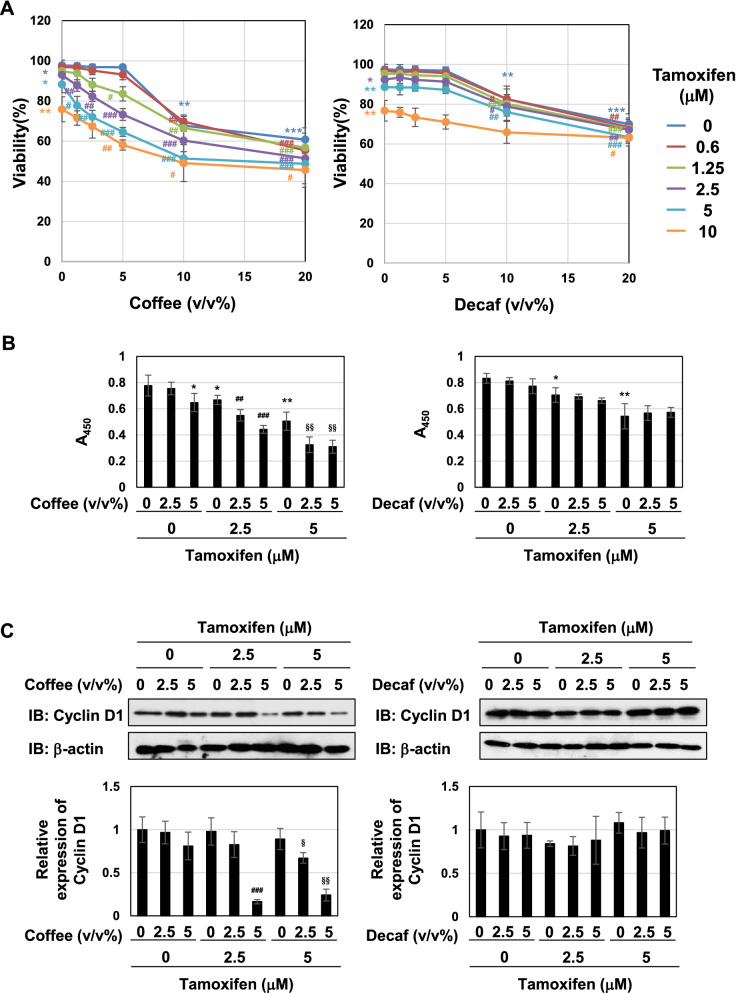

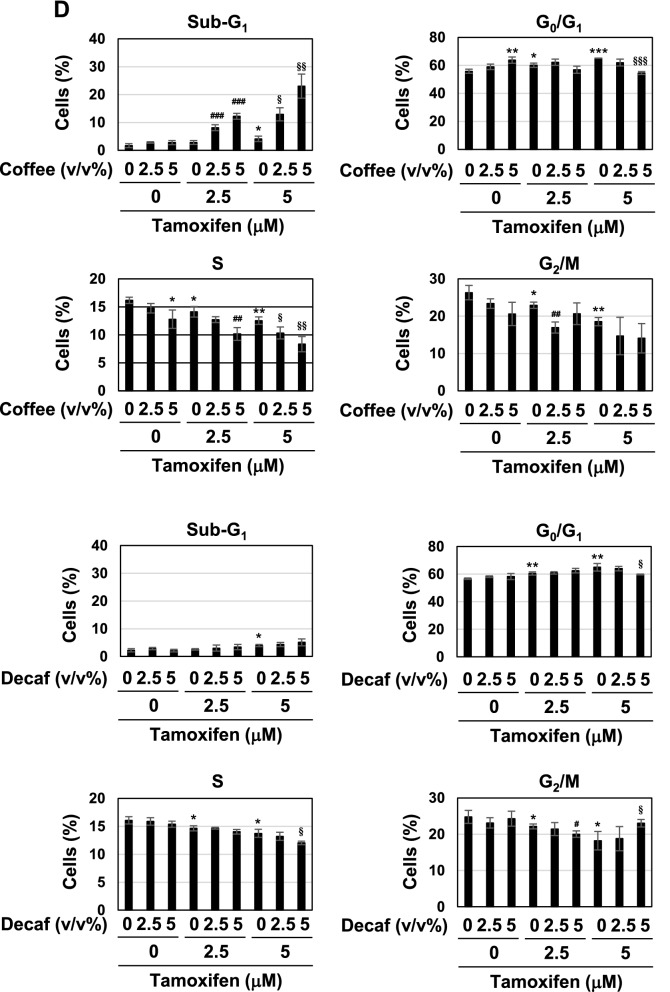

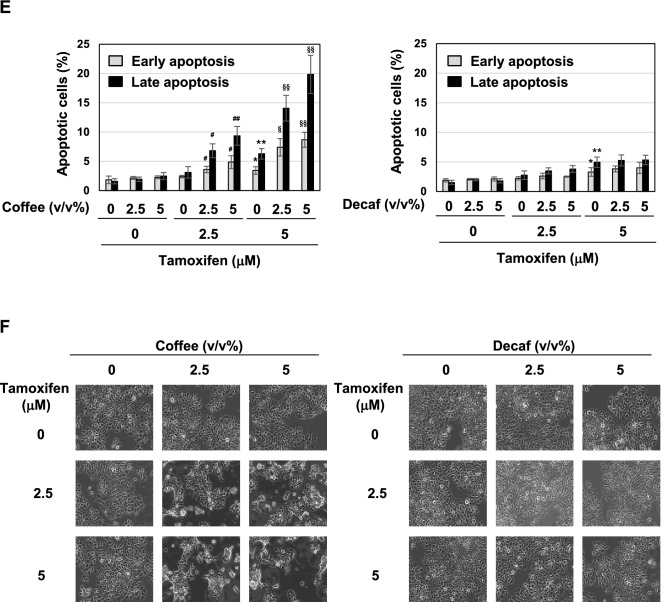
Co-treatment of coffee with tamoxifen suppresses cell proliferation and induces apoptosis in MCF-7 cells. (**A**) MCF-7 cells were treated with coffee or decaffeinated coffee (1.25, 2.5, 5, 10, 20 v/v%) in the presence or absence of tamoxifen (0.6, 1.25, 2.5, 5, 10 μM) for 24 h. Cell viability was measured by the trypan blue exclusion method. Results represent the mean ± SD of three independent experiments. *p < 0.05, **p < 0.01, ***p < 0.001 significantly different from control cells. ^#^*p* < 0.05, ^##^*p* < 0.01, ^###^*p* < 0.001 significantly different from cells treated with tamoxifen. (**B**–**D**, **F**) MCF-7 cells were treated with coffee or decaffeinated coffee (2.5, 5 v/v%) in the presence of tamoxifen (2.5, 5 μM) for 24 h. (**B**) The proliferation rate was determined by BrdU incorporation assay. Results represent the mean ± SD of four independent experiments. *p < 0.05, **p < 0.01 significantly different from control cells, ^##^*p* < 0.01, ^###^*p* < 0.001 significantly different from cells treated with 2.5 μM tamoxifen and ^§§^*p* < 0.01 significantly different from cells treated with 5 μM tamoxifen. (**C**) Whole cell lysates were immunoblotted with an anti-cyclin D1 antibody or anti-β-actin antibody. The relative expression levels of cyclin D1 are shown in the graphs. Results represent the mean ± SD of three independent experiments. ^###^*p* < 0.001 significantly different from cells treated with 2.5 μM tamoxifen. ^§^*p* < 0.05, ^§§^*p* < 0.01 significantly different from cells treated with 5 μM tamoxifen. (**D**) Cells were fixed, permeabilized and treated with propidium iodide, and the cell cycle was examined using a flow cytometric analysis. The ratios of cells in the Sub-G_1_ phase, G_0_/G_1_ phase, S phase and G_2_/M phase were graphed. Data were expressed as means ± SD of three independent experiments. *p < 0.05, **p < 0.01, *** *p* < 0.001 significantly different from control cells. ^#^*p* < 0.05, ^##^*p* < 0.01, ^###^*p* < 0.001 significantly different from cells treated with 2.5 μM tamoxifen. ^§^*p* < 0.05, ^§§^*p* < 0.01, ^§§§^*p* < 0.001 significantly different from cells treated with 5 μM tamoxifen. (E) MCF-7 cells were treated with coffee or decaffeinated coffee (2.5, 5v/v%) in the presence of tamoxifen (2.5, 5 μM) for 18 h. Annexin V–FITC and propidium iodide (PI) double staining was performed. The ratios of early-phase apoptotic cells and late-phase apoptotic cells were graphed. Data were expressed as means ± SD (n = 3). *p < 0.05, **p < 0.01 significantly different from control cells. ^#^*p* < 0.05, ^##^*p* < 0.01 significantly different from cells treated with 2.5 μM tamoxifen. ^§^*p* < 0.05, ^§§^*p* < 0.01 significantly different from cells treated with 5 μM tamoxifen. (**F**) Morphological changes of MCF-7 cells were viewed using a brightfield microscope under × 40 magnification.

To elucidate the molecular mechanisms by which coffee decoction enhances the anti-cancer effects of tamoxifen against breast cancer, we selected the breast cancer-derived cell line, MCF-7 because it expresses ERα^33^. We prepared the coffee decoctions from of commercially available roasted coffee beans and decaffeinated coffee beans, respectively. And then, we first investigated the effects of various concentrations of these coffee decoctions on the viability of MCF-7 cells. The treatment with more than 10 v/v% coffee decoction and decaffeinated coffee decoction significantly reduced the viability of MCF-7 cells, however; less than 5 v/v% coffee decoction and decaffeinated coffee decoction had no effect on the viability. Next, we attempted the effects of co-treatment with coffee decoction and tamoxifen. The treatment with more than 2.5 μM tamoxifen alone significantly reduced the viability of MCF-7 cells. Interestingly, on the condition that 1.25 μM tamoxifen was co-treated, 5 v/v% or less of coffee decoction markedly reduced the viability of MCF-7 cells. On the other hand, decaffeinated coffee decoction failed to exhibit the comparable effects on cell viability (Fig. 1A). The viability of MCF-7 cells was significantly reduced by 24 h co-treatment with coffee (5 v/v%) and tamoxifen (2.5, 5 μM) (Supplementary Fig. S1). We calculated combination index (CI) to evaluate whether the activity of coffee and tamoxifen to induce cell death in MCF-7 cells is synergistic or additive. Since a CI of less than 1, equal to 1, and more than 1 indicates synergy, additivity, and antagonism, respectively^34^, it was shown that the combination with tamoxifen at 1.25 μM, 2.5 μM and 5 μM and less than 5 v/v% coffee decoction synergistically induced cell death in MCF-7 cells (Table 1). We also determined the effect of coffee and decaffeinated coffee with/without tamoxifen on cell proliferation of MCF-7 cells by BrdU incorporation assay. The treatment with 5 v/v% coffee decoction alone and tamoxifen alone reduced the cell proliferation. Whereas the co-treatment with coffee decoction and tamoxifen significantly reduced the cell proliferation, the co-treatment with decaffeinated did not reduce the proliferation of cells treated with tamoxifen (Fig. 1B). Cyclin D1 functions as a driver of cell-cycle progression from the G_1_ to S phases, and is a suitable marker protein for cell-cycle progression^35^. The co-treatment with coffee and tamoxifen down-regulated cyclin D1 expression in a dose-dependent manner (Fig. 1C, left), and decaffeinated coffee failed to exert similar effects (Fig. 1C, right). We then investigated alterations in the cell-cycle population. As shown in Fig. 1D, more than 2.5 μM tamoxifen increased the percentage of cells in G_0_/G_1_ phase and reduced the those in the S phase and the G_2_/M phase and 5 μM tamoxifen increased the percentage of cells in the sub-G_1_ phase, which is consist with cell death^36^. Whereas 5 v/v% coffee decoction significantly increased the percentage of cells in the G_0_/G_1_ phase and reduced those in the S phase, decaffeinated coffee decoction had no effect on cell-cycle population. The co-treatment with more than 2.5 μM coffee decoction and tamoxifen effectively reduced the percentage of cells in the S phase and markedly increased those in the sub-G1 phase. On the other hand, the co-treatment with 5 μM decaffeinated coffee decoction and 5 μM tamoxifen reduced the percentage of cells in the S phase (Fig. 1D). We also performed Annexin V-PI assay to detect early-phase apoptotic cells and late-stage apoptotic cells. Under the condition that tamoxifen was co-presence, coffee but not decaffeinated coffee drastically increased the ratio of apoptosis in a dose-dependent manner (Fig. 1E). After the co-treatment with coffee and tamoxifen, MCF-7 cells showed a marked change in morphology, including shrinkage and irregular shape. On the other hand, the combination of decaffeinated coffee and tamoxifen had little effect on morphological change of MCF-7 cells (Fig. 1F).

**Table 1:** Combination index (CI).

	Conc (mM)	CI
Tamoxifen	0.6	2.11
	1.25	0.78
	2.5	0.71
	5	0.92
	10	1.45

### Coffee decoction suppresses ERα expression and its transcriptional activity

**Figure 2:**
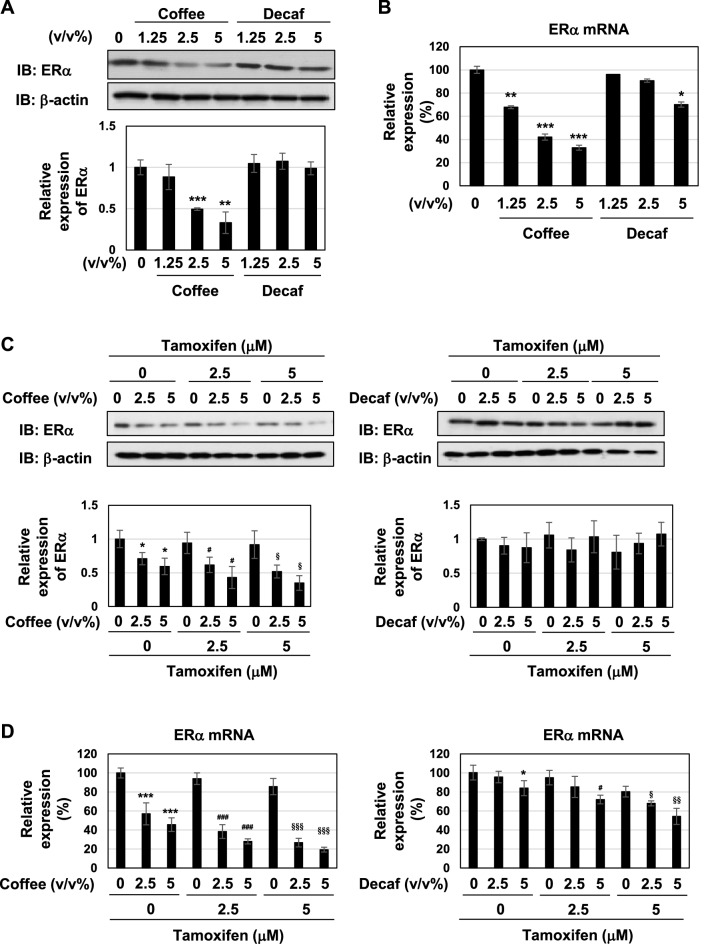
Coffee, but not decaffeinated coffee, inhibits ERα expression in MCF-7 cells. (**A**, **B**) MCF-7 cells were treated with various concentrations of coffee and decaffeinated coffee (1.25, 2.5, 5 v/v%) for 24 h. (**C**, **D**) MCF-7 cells were treated with various concentrations of coffee and decaffeinated coffee (2.5, 5 v/v%) in the absence or presence of tamoxifen (2.5, 5 μM) for 24 h. (**A**, **C**) Whole cell lysates were immunoblotted with an anti-ERα antibody or anti-β-actin antibody. The relative expression levels of ERα are shown in the graphs. Results represent the mean ± SD of three independent experiments. *p < 0.05, **p < 0.01, ***p < 0.001 significantly different from control cells. ^#^*p* < 0.05, ^§^*p* < 0.05 significantly different from cells treated with 2.5 μM tamoxifen and 5 μM tamoxifen, respectively. (**B**, **D**) The expression of ERα mRNA was assessed by an RT-PCR analysis. The expression of GAPDH mRNA was used as an internal control. Values are given as the mean ± SD of three independent experiments. *p < 0.05, **p < 0.01, ***p < 0.001 significantly different from control cells. ^#^*p* < 0.05, ^###^*p* < 0.001 significantly different from cells treated with 2.5 μM tamoxifen. ^§^*p* < 0.05, ^§§^*p* < 0.01, ^§§§^*p* < 0.001 significantly different from cells treated with 5 μM tamoxifen.

The effects of coffee decoction on the expression of ERα were examined. The treatment with coffee but not decaffeinated coffee effectively decreased the expression of the ERα protein in a dose-dependent manner (Fig. 2A), and this appeared to be due to reductions in ERα mRNA levels (Fig. 2B). Whereas coffee decoction markedly reduced the expression of ERα protein and ERα mRNA regardless of the treatment with tamoxifen, the combination of decaffeinated coffee and tamoxifen had no effect on the expression of ERα protein and reduced the expression of ERα mRNA (Fig. 2C,D).

**Figure 3:**
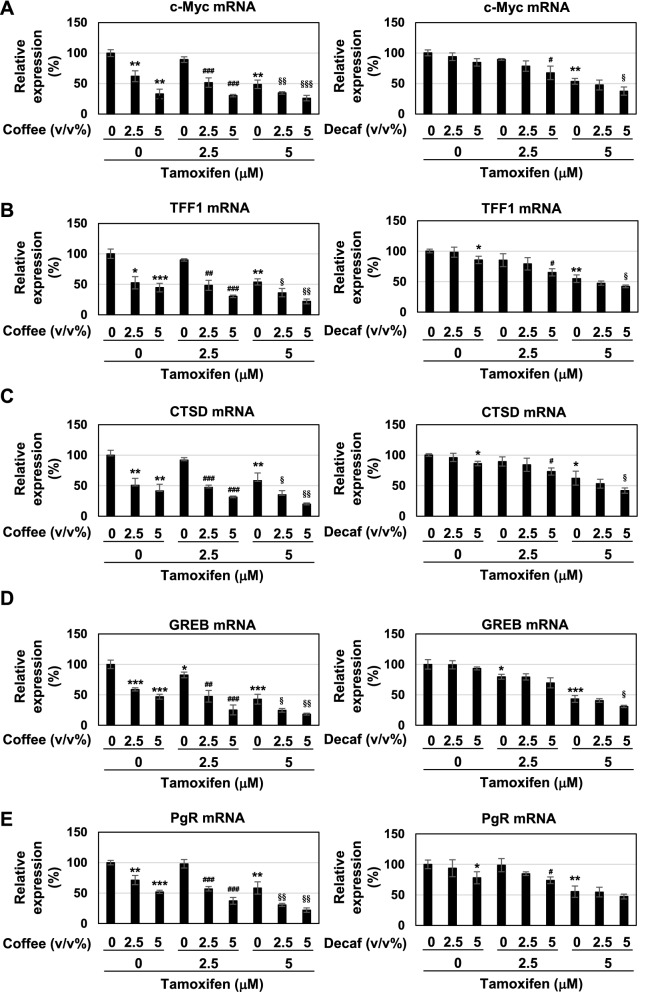
Coffee, but not decaffeinated coffee, inhibits the expression of ERα-target genes in MCF-7 cells. MCF-7 cells were treated with various concentrations of coffee and decaffeinated coffee (2.5, 5 v/v%) in the presence of tamoxifen (2.5, 5 μM) for 24 h. The mRNA expression of c-Myc, TFF1, CTSD, GREB, and PgR was assessed by an RT-PCR analysis. The expression of GAPDH mRNA was used as an internal control. Values are given as the mean ± SD of three independent experiments. *p < 0.05, **p < 0.01, ***p < 0.001 significantly different from control cells. ^#^*p* < 0.05, ^##^*p* < 0.01, ^###^*p* < 0.001 significantly different from cells treated with 2.5 μM tamoxifen. ^§^*p* < 0.05, ^§§^*p* < 0.01, ^§§§^*p* < 0.001 significantly different from cells treated with 5 μM tamoxifen.

We investigated the effects of coffee decoction on the transcriptional activity of ERα. Once stimulated with estrogen, ERα has been shown to directly induce the expression of several target genes, such as c-Myc, TFF1, CTSD, GREB, and PgR^6–10^. Quantitative PCR was performed to examine the effects of coffee decoction and tamoxifen on the mRNA expression levels of these target genes of ERα. As shown in Fig. 3A–E (each left graph), coffee decoction alone and tamoxifen alone reduced the expression of the ERα target genes and the co-treatment with coffee decoction (2.5, 5 v/v%) induced further reductions on their expression in cells treated with tamoxifen. On the other hand, 5 v/v% decaffeinated coffee reduced the expression of TFF1 mRNA, CTSD mRNA and PgR mRNA in MCF-7 cells and further reduced the expression of c-Myc mRNA, TFF1 mRNA, CTSD mRNA and GREB mRNA in MCF-7 cells treated with 5 μM tamoxifen as shown in Fig. 3A–E (each right graph). These results suggest that coffee decoction and tamoxifen inhibit the activity of ERα by reducing the expression of ERα mRNA and antagonizing ERα, respectively.

### Coffee decoction and tamoxifen cooperatively stimulate the activation of the p53 tumor suppressor

**Figure 4:**
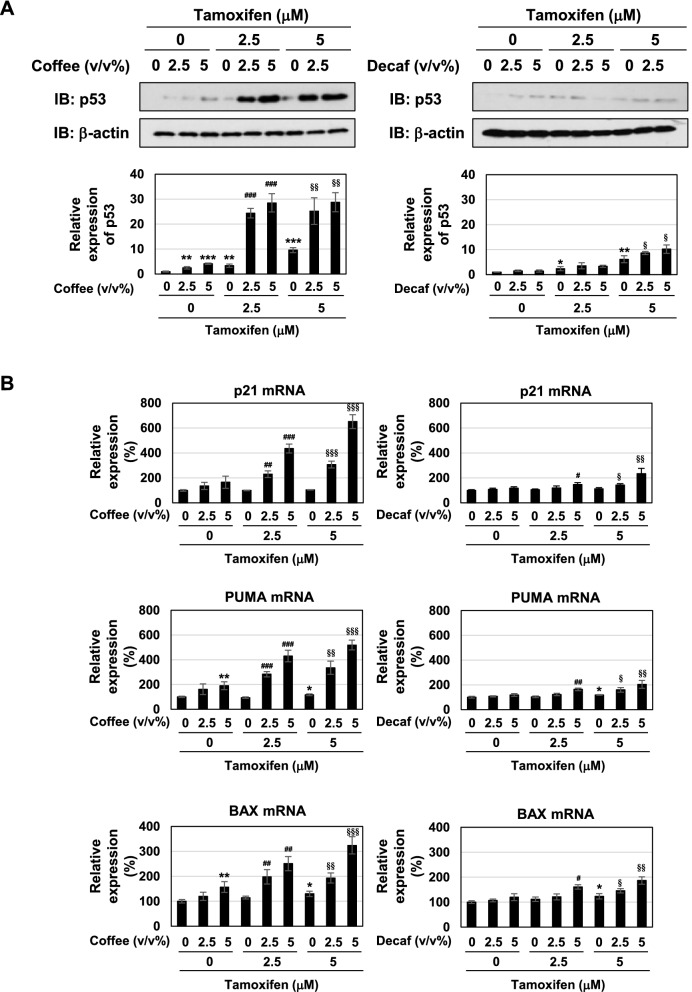
Coffee, but not decaffeinated coffee, cooperates with tamoxifen to activate p53 in MCF-7 cells. MCF-7 cells were treated with coffee or decaffeinated coffee (2.5, 5 v/v%) in the absence or presence of tamoxifen (2.5, 5 μM) for 24 h. (**A**) Whole cell lysates were immunoblotted with an anti-p53 antibody or anti-actin antibody. The relative expression levels of p53 are shown in the graphs. Results represent the mean ± SD of three independent experiments. *p < 0.05, **p < 0.01, ***p < 0.001 significantly different from control cells. ^###^*p* < 0.001 significantly different from cells treated with 2.5 μM tamoxifen. ^§^*p* < 0.05, ^§§^*p* < 0.01 significantly different from cells treated with 5 μM tamoxifen. (**B**) The expression of p21 mRNA, PUMA mRNA, and BAX mRNA was assessed by an RT-PCR analysis. The expression of GAPDH mRNA was used as an internal control. Values are given as the mean ± SD of three independent experiments. *p < 0.05, **p < 0.01 significantly different from control cells. ^#^*p* < 0.05, ^##^*p* < 0.01, ^###^*p* < 0.001 significantly different from cells treated with 2.5 μM tamoxifen. ^§^*p* < 0.05, ^§§^*p* < 0.01, ^§§§^*p* < 0.001 significantly different from cells treated with 5 μM tamoxifen.

The p53 tumor suppressor induces cell-cycle arrest and apoptotic cell death through the expression of its target genes, such as p21^Cip1^, PUMA, and BAX^37–39^. To clarify the mechanisms by which coffee decoction and tamoxifen induce cell-cycle arrest and apoptosis, we examined their effects on the activation of the p53 tumor suppressor. As shown in Fig. 4A, coffee decoction alone and 5 μM tamoxifen alone induced the accumulation of p53. The accumulation of p53 induced by coffee decoction was markedly enhanced by tamoxifen (Fig. 4A, left). The effect of decaffeinated coffee decoction to induce p53 expression in MCF-7 cells treated with tamoxifen was considerably small in comparison with the effect of coffee decoction (Fig. 4A, right). We then investigated the effects of coffee decoction and tamoxifen on the expression of the target genes of p53. As shown in Fig. 4B, the mRNA expression of PUMA and BAX was induced by coffee decoction, but not decaffeinated coffee. The treatment with 5 μM tamoxifen alone induced the mRNA expression of PUMA and BAX. Furthermore, the co-treatment with coffee decoction and tamoxifen markedly increased the expression of p21^Cip1^, PUMA, and BAX. Although the reinforcement effect of decaffeinated coffee was smaller than that of coffee decoction, but decaffeinated coffee decoction enhanced mRNA expression of p21^Cip1^, PUMA, and BAX in MCF-7 cells treated with tamoxifen (Fig. 4B).

We confirmed that the decoction of coffee which we prepared by roasting green coffee beans also induced cell death, downregulation of ERα and expression of p53 in MCF-7 cells treated with tamoxifen as well as decoction of purchased coffee (Supplementary Fig. S2). Therefore, these results suggest that coffee decoction enhanced the anti-tumor activity of tamoxifen by inducing p53 activation.

### Coffee decoction and tamoxifen cooperatively induce cell death and p53 expression specifically in MCF-7 cells

**Figure 5:**
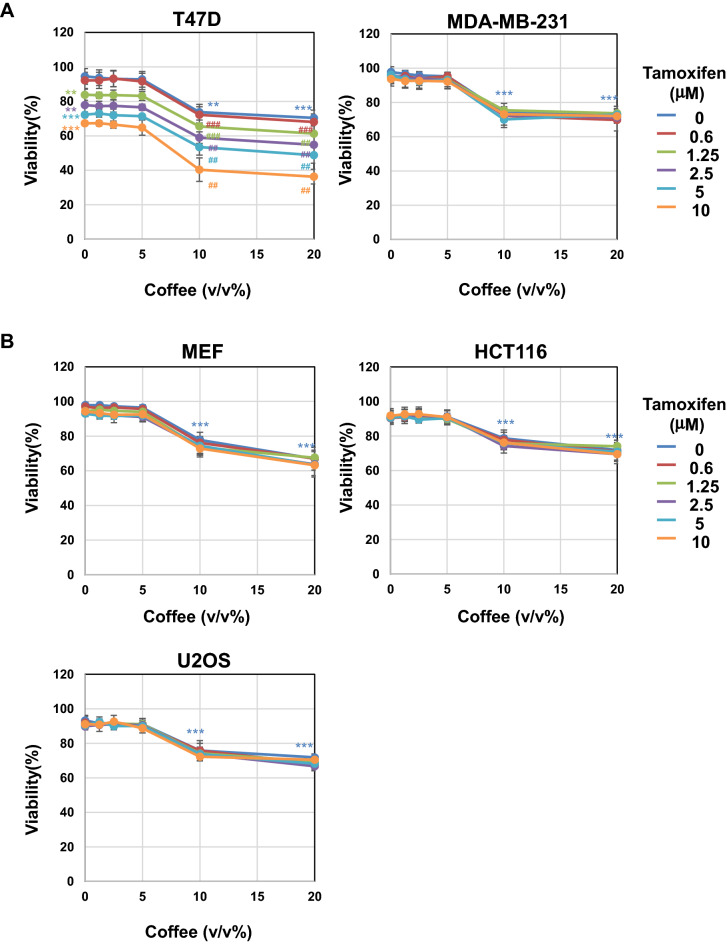

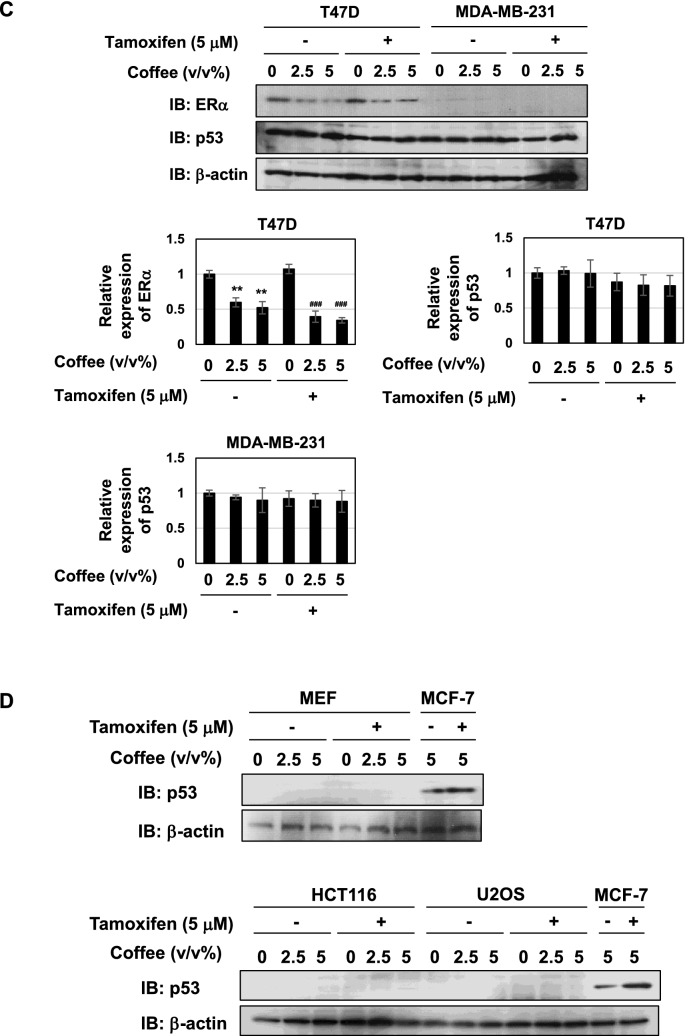
Co-treatment of coffee with tamoxifen specifically induces cell death only in MCF-7 cells. (**A**) T47D cells and MDM-MB-231 cells and (**B**) Murine embryonic fibroblast cells (MEF), HCT116 cells and U2OS cells were treated with coffee (1.25, 2.5, 5, 10, 20 v/v%) in the absence or presence of tamoxifen (0.6, 1.25, 2.5, 5, 10 μM) for 24 h. Cell viability was measured by the trypan blue exclusion method. Results represent the mean ± SD of three independent experiments. **p < 0.01, ***p < 0.001 significantly different from control cells. ^##^*p* < 0.01, ^###^*p* < 0.001 significantly different from cells treated with tamoxifen. (**C**, **D**) T47D cells, MDM-MB-231 cells, MEF, HCT116 cells and U2OS cells were treated with coffee (2.5, 5 v/v%) and MCF-7 cells were treated with coffee (5 v/v%) in the absence and presence of tamoxifen (5 μM) for 24 h. Whole cell lysates were immunoblotted with an anti-ERα antibody, anti-p53 antibody or anti-β-actin antibody. The relative expression levels of ERα and p53 are shown in the graphs. Results represent the mean ± SD of three independent experiments. **p < 0.01, ^###^*p* < 0.001 significantly different from control cells and cells treated with 5 μM tamoxifen, respectively.

In order to investigate the mechanism how the co-treatment with coffee decoction and tamoxifen induce apoptosis in MCF-7 cells, we examined their effect on the viability of other breast cancer cell lines such as T47D and MDA-MB-231. T47D are ERα-positive cells and MDA-MB-231 are ERα-negative cells, respectively, and these two cells express mutant p53^40,41^. More than 10 v/v% coffee decoction reduced the viability of T47D cells and MDA-MB-231 cells, but 5 v/v% or less coffee decoction had no effect on their viability. Although the treatment with tamoxifen reduced the viability of T47D in a dose-dependent manner, the co-treatment of coffee decoction did not enhance the reduction in viability of T47D cells treated with tamoxifen (Fig. 5A, left). On the other hand, the co-treatment with 5 v/v% or less coffee decoction and tamoxifen had no effect on the viability of MDA-MB-231 cells (Fig. 5A, right). Furthermore, we also examined the effect of coffee and tamoxifen on the viability of non-tumor normal cells such as murine embryonic cells (MEF) and two kinds of tumor cells harboring wild type p53 such as HTC116 cells and U2OS cells^42,43^. More than 10 v/v% coffee decoction exhibited cytotoxicity to all cells but 5 v/v% or less coffee decoction had no effect on the viability of MEF, HTC116 cells and U2OS cells. Furthermore, the co-treatment of 5 v/v% or less coffee decoction and tamoxifen had no effect on the viability of these cells (Fig. 5B). We next investigated the effect of coffee decoction and tamoxifen on the expression level of ERα and p53 in T47D cells and MDA-MB-231 cells. The treatment with coffee decoction significantly reduced the expression of ERα regardless of the treatment with tamoxifen in T47D cells. Although p53 was expressed in T47D cells and MDA-MB-231 cells, the treatment with coffee decoction and tamoxifen did not affect its expression level (Fig. 5C). In addition, the co-treatment with coffee decoction and tamoxifen failed to induce p53 expression in MEF, HCT116 and U2OS (Fig. 5D). These results suggest that the enhancement of tamoxifen proapoptotic activity induced by coffee decoction was observed only in ER-positive and p53 wild-type MCF-7 cells.

### Coffee decoction may induce p53-mediated apoptosis by suppressing ERK and PI3K pathways

**Figure 6:**
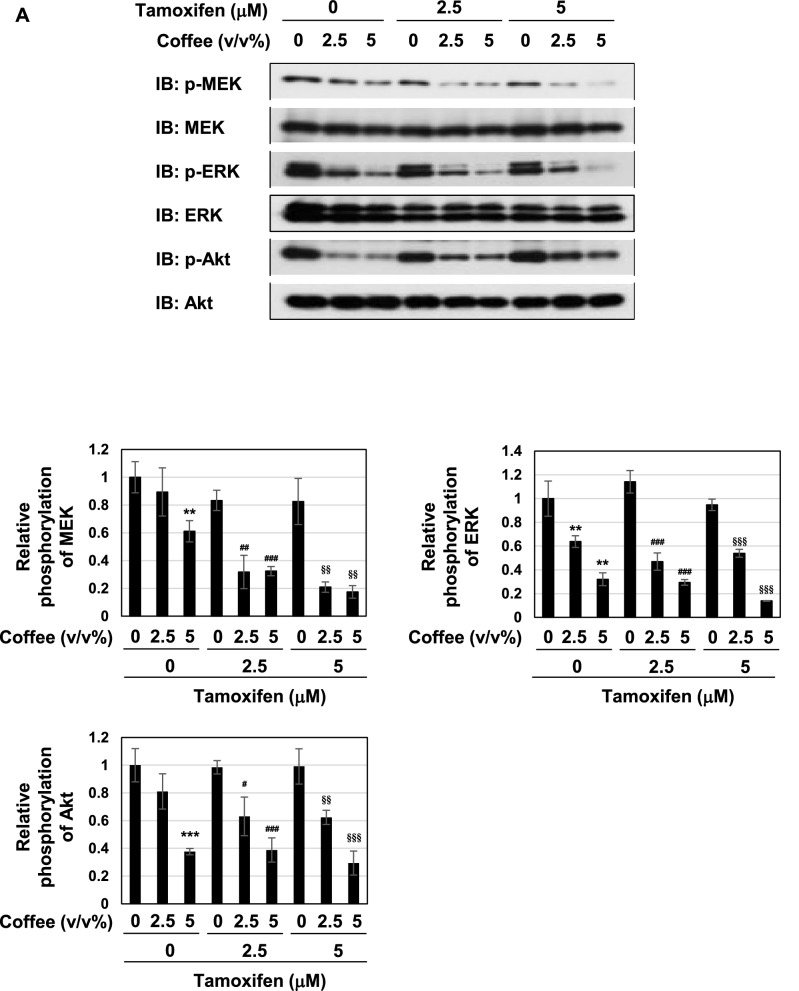

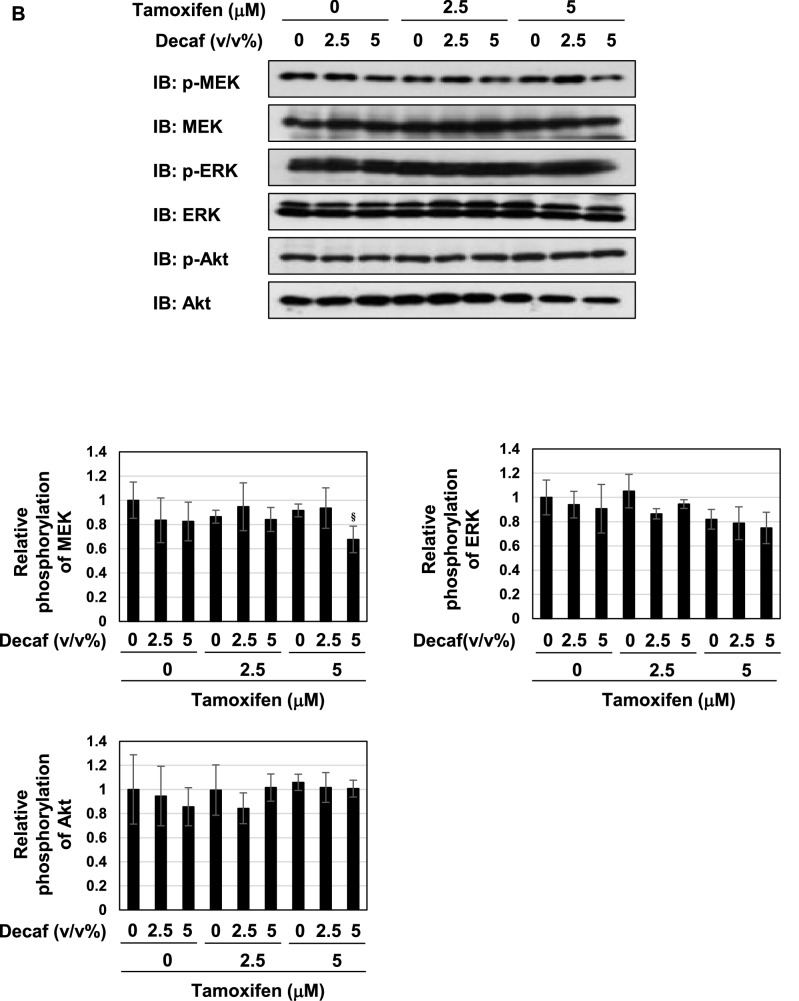
Coffee, but not decaffeinated coffee inhibits the MEK-ERK pathway and Akt activation regardless of tamoxifen in MCF-7 cells. (**A**, **B**) MCF-7 cells were treated with coffee (2.5, 5 v/v%) or decaffeinated coffee (2.5, 5 v/v%) in the absence or presence of tamoxifen (2.5, 5 μM) for 24 h. Whole cell lysates were immunoblotted with an anti-phospho-MEK, anti-MEK, anti-phospho-ERK, anti-ERK, anti-phospho-Akt, or anti-Akt antibody. The relative phosphorylation levels of MEK, ERK and Akt are shown in the graphs. Results represent the mean ± SD of three independent experiments. **p < 0.01, ***p < 0.001 significantly different from control cells. ^#^*p* < 0.05, ^##^*p* < 0.01, ^###^*p* < 0.001 significantly different from cells treated with 2.5 μM tamoxifen. ^§^*p* < 0.05, ^§§^*p* < 0.01, ^§§§^*p* < 0.001 significantly different from cells treated with 5 μM tamoxifen.

We were also interested in the effects of coffee decoction on cell proliferative and survival signals, such as MAP kinases and Akt^44,45^. Therefore, we investigated the effects of coffee decoction on the phosphorylation of ERK, a major member of MAP kinase family, and MEK, an upstream kinase of ERK and Akt. As shown in Fig. 6A, coffee decoction alone exerted potent inhibitory effects on the phosphorylation of MEK, ERK and Akt. On the other hand, decaffeinated coffee did not exert these inhibitory effects (Fig. 6B).

**Figure 7:**
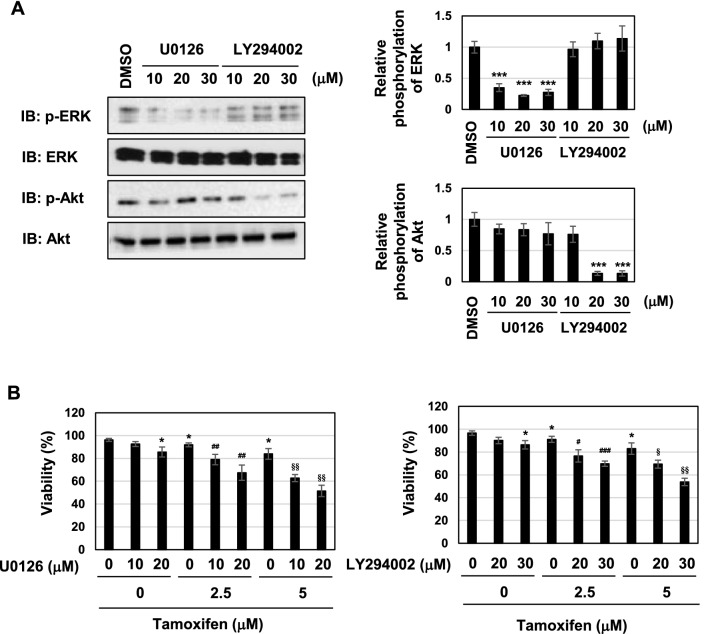

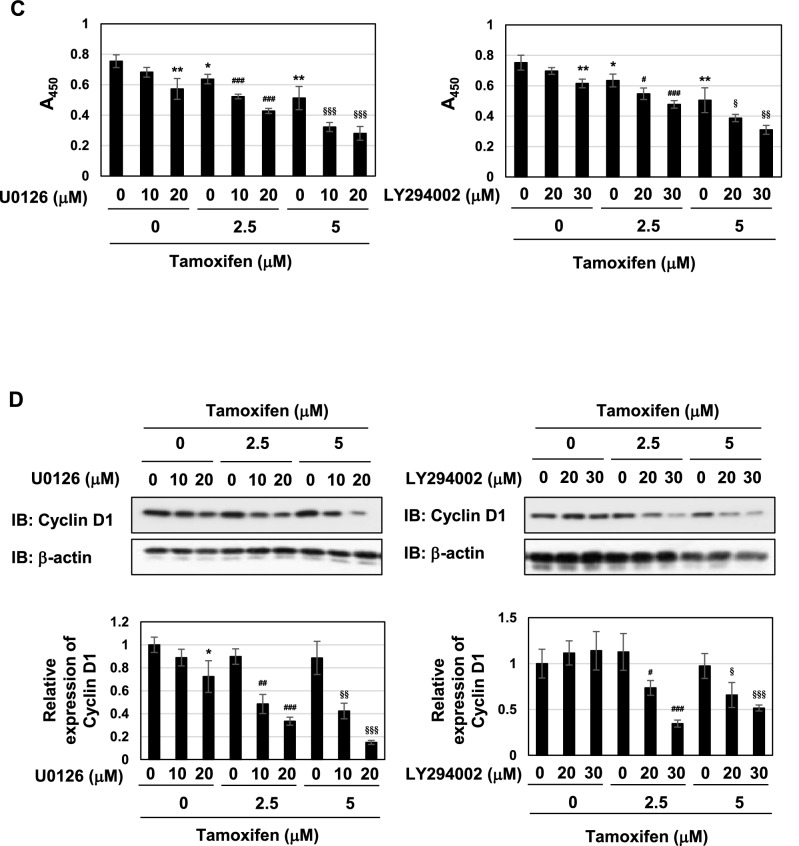

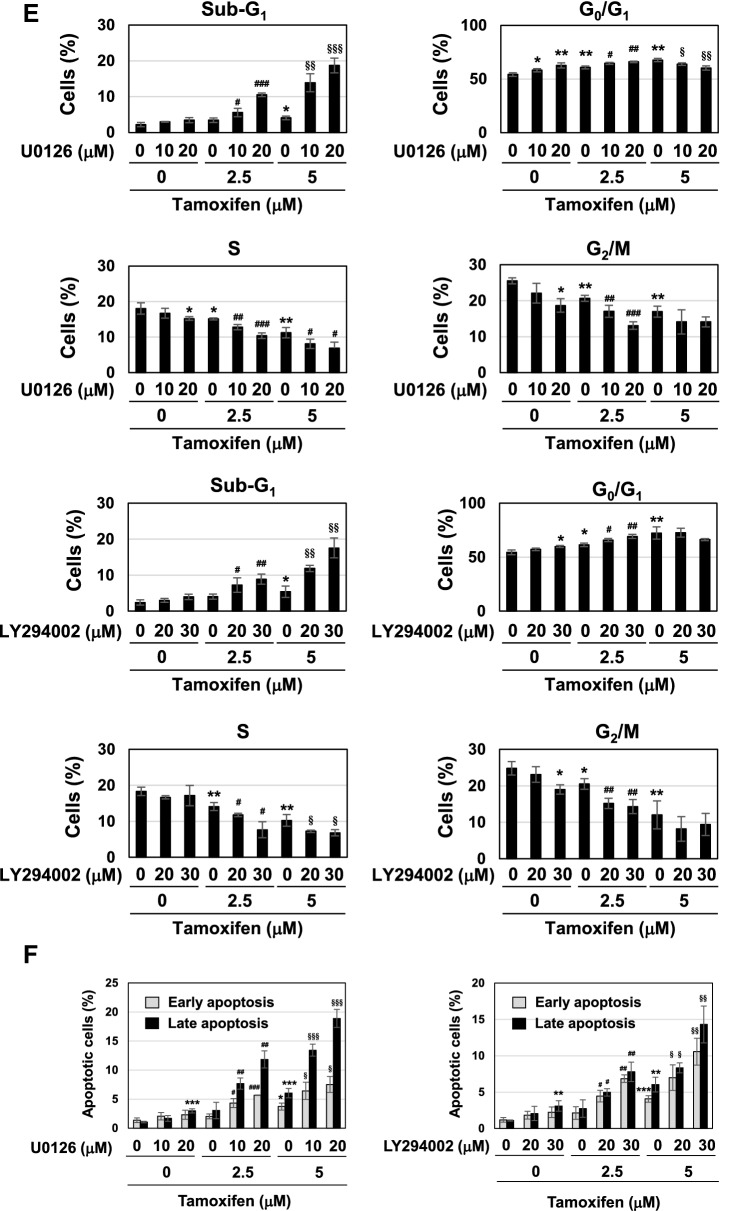
U0126 and LY294002 induce apoptosis in the presence of tamoxifen in MCF-7 cells. (**A**) MCF-7 cells were treated with DMSO (0.1%), U0126 (10, 20, 30 μM), or LY294002 (10, 20, 30 μM) for 24 h. Whole cell lysates were immunoblotted with anti-phospho-ERK, anti-ERK, anti-phospho-Akt, or anti-Akt. The relative phosphorylation levels of ERK and Akt are shown in the graphs. Results represent the mean ± SD of three independent experiments. ***p < 0.001 significantly different from control cells. (B-E) MCF-7 cells were treated with DMSO (0.1%), U0126 (10, 20 μM), or LY294002 (20, 30 μM) in the absence or presence of tamoxifen (2.5, 5 μM) for 24 h. (**B**) Cell viability was measured by the trypan blue exclusion method. Results represent the mean ± SD of three independent experiments. *p < 0.05 significantly different from control cells. ^#^*p* < 0.05, ^##^*p* < 0.01, ^###^*p* < 0.001 significantly different from cells treated with 2.5 μM tamoxifen. ^§^*p* < 0.05, ^§§^*p* < 0.01 significantly different from cells treated with 5 μM tamoxifen. (**C**) The proliferation rate was determined by BrdU incorporation assay. Results represent the mean ± SD of four independent experiments. *p < 0.05, **p < 0.01 significantly different from control cells. ^#^*p* < 0.05, ^###^*p* < 0.001 significantly different from cells treated with 2.5 μM tamoxifen. ^§^*p* < 0.05, ^§§^*p* < 0.01, ^§§§^*p* < 0.001 significantly different from cells treated with 5 μM tamoxifen. (**D**) Whole cell lysates were immunoblotted with an anti-cyclin D1 antibody or anti-β-actin antibody. The relative expression levels of cyclin D1 are shown in the graphs. Results represent the mean ± SD of three independent experiments. *p < 0.05 significantly different from control cells. ^#^*p* < 0.05, ^##^*p* < 0.01, ^###^*p* < 0.001 significantly different from cells treated with 2.5 μM tamoxifen. ^§^*p* < 0.05, ^§§^*p* < 0.01, ^§§§^*p* < 0.001 significantly different from cells treated with 5 μM tamoxifen. (**E**) Cells were fixed, permeabilized and treated with propidium iodide fixed, treated with propidium iodide, and the cell cycle was examined using a flow cytometric analysis. The ratios of cells in the Sub-G_1_ phase, G_0_/G_1_ phase, S phase and G_2_/M phase were graphed. Data were expressed as means ± SD of three independent experiments. *p < 0.05, **p < 0.01 significantly different from control cells. ^#^*p* < 0.01, ^##^*p* < 0.05, ^###^*p* < 0.001 significantly different from cells treated with 2.5 μM tamoxifen. ^§^*p* < 0.05, ^§§^*p* < 0.01, ^§§§^*p* < 0.001 significantly different from cells treated with 5 μM tamoxifen. (**F**) MCF-7 cells were treated with DMSO (0.1%), U0126 (10, 20 μM), or LY294002 (20, 30 μM) in the absence or presence of tamoxifen (2.5, 5 μM) for 18 h. Annexin V–FITC and propidium iodide (PI) double staining was performed. The ratios of early-phase apoptotic cells and late-phase apoptotic cells were graphed. Data were expressed as means ± SD of three independent experiments. *p < 0.05, **p < 0.01, ***p < 0.001 significantly different from control cells. ^#^*p* < 0.05, ^##^*p* < 0.01, ^###^*p* < 0.001 significantly different from cells treated with 2.5 μM tamoxifen. ^§^*p* < 0.05, ^§§^*p* < 0.01, ^§§§^*p* < 0.001 significantly different from cells treated with 5 μM tamoxifen.

These results prompted us to investigate whether coffee decoction-induced cell-cycle arrest and apoptosis may be attributed to the inhibition of the ERK and Akt pathways. U0126 and LY294002 are inhibitors of MEK and PI3K, and these compounds inhibited the activation of their downstream molecules, such as ERK and Akt (Fig. 7A). The ERK and Akt pathways contribute to cell proliferation by mainly functioning as downstream signals of Ras small GTPase^44,46^. The treatment with U0126 alone reduced the viability and suppressed cell proliferation and the expression of cyclin D1, and the treatment with LY294002 alone reduced the viability and suppressed cell proliferation in MCF-7 cells (Fig. 7B–D). These inhibitors and tamoxifen cooperatively reduced the viability and suppressed cell proliferation and expression level of cyclin D1 in MCF-7 cells (Fig. 7B–D). The treatment with U0126 alone increased the percentage of cells in the G_0_/G_1_ phase and reduced those in the S phase and G_2_/M phase, and the co-treatment with U0126 with tamoxifen markedly increased the percentage of cells in the sub-G_1_ phase and reduced those in the S phase. The treatment with LY294002 alone increased the percentage of cells in the G_0_/G_1_ phase and reduced those in the G_2_/M phase, and the co-treatment with LY294002 with tamoxifen markedly increased the percentage of cells in the sub-G_1_ phase and reduced those in the S phase (Fig. 7E). Furthermore, the co-treatment with U0126 or LY294002 and tamoxifen markedly induced apoptosis in MCF-7 cells (Fig. 7F).

**Figure 8:**
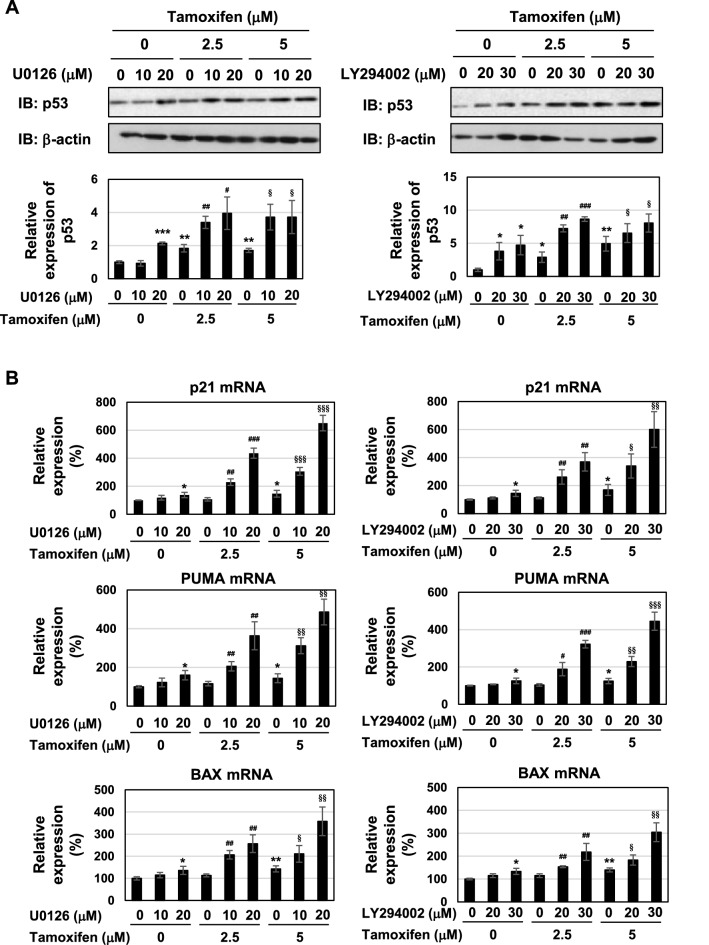
U0126 and LY294002 with tamoxifen induce p53 activation in MCF-7 cells. MCF-7 cells were treated with DMSO (0.1%), U0126 (10, 20 μM), or LY294002 (20, 30 μM) in the presence of tamoxifen (2.5, 5 μM) for 24 h. (**A**) Whole cell lysates were immunoblotted with an anti-p53 antibody or anti-β-actin antibody. The relative expression levels of p53 are shown in the graphs. Results represent the mean ± SD of three independent experiments. *p < 0.05, **p < 0.01, ***p < 0.001 significantly different from control cells. ^#^*p* < 0.05, ^##^*p* < 0.01, ^###^*p* < 0.001 significantly different from cells treated with 2.5 μM tamoxifen. ^§^*p* < 0.05 significantly different from cells treated with 5 μM tamoxifen. (**B**) The mRNA expression of p21, PUMA, and BAX was assessed by an RT-PCR analysis. The expression of GAPDH mRNA was used as an internal control. Values are given as the mean ± SD of three independent experiments. *p < 0.05, **p < 0.01 significantly different from control cells. ^#^*p* < 0.05, ^##^*p* < 0.01, ^###^*p* < 0.001 significantly different from cells treated with 2.5 μM tamoxifen. ^§^*p* < 0.05, ^§§^*p* < 0.01, ^§§§^*p* < 0.001 significantly different from cells treated with 5 μM tamoxifen.

To gain further insights into the mechanisms by which the suppression of the ERK or Akt pathway induced cell-cycle arrest and apoptosis, we examined the p53 pathway. The treatment with tamoxifen, U0126 or LY294002 alone induced the accumulation of p53 and expression of p53 target genes, such as p21^Cip1^, PUMA, and BAX in MCF-7 cells (Fig. 8A,B). Furthermore, in the presence of tamoxifen, co-treatment with U0126 or LY294002 caused further accumulation of p53 protein and the increased expression of p53 target genes (Fig. 8A,B). According to the results shown in Figs. 6, 7 and 8, the combination of tamoxifen and coffee decoction appeared to induce cell-cycle arrest and apoptotic cell death, which may have been mediated by the activation of p53, and this appeared to be through the inhibition of the ERK or Akt pathway.

### Caffeine exhibits the ability to reduce the expression of ERα and its transcriptional activity but does not induce apoptosis in MCF-7 cells

**Figure 9:**
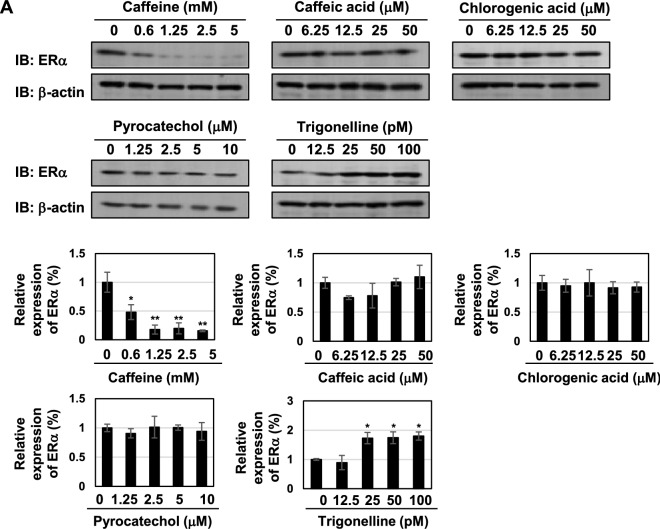

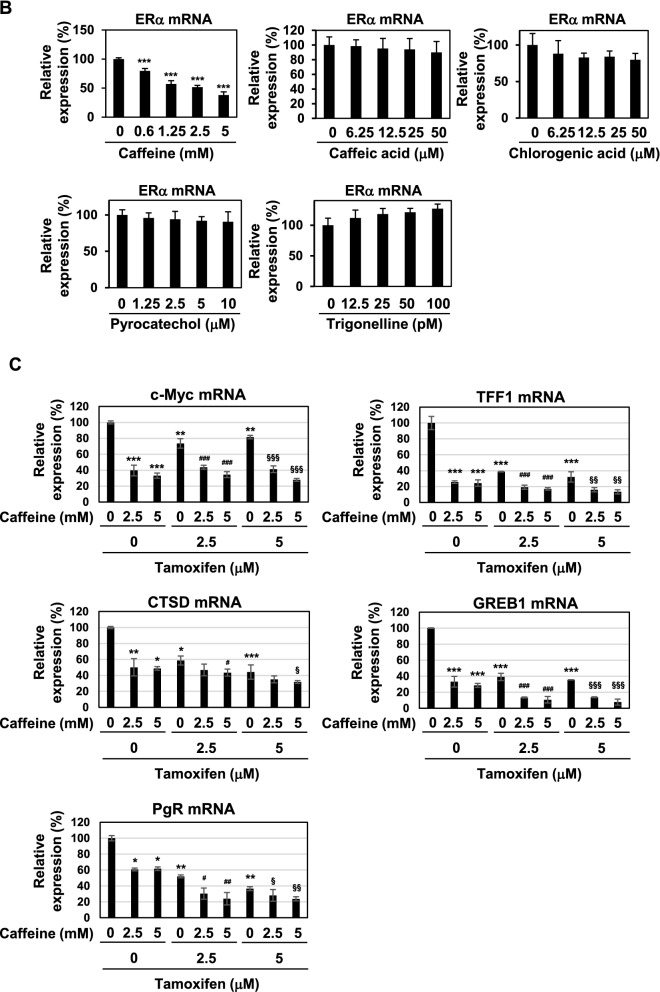
Caffeine inhibits ERα expression in MCF-7 cells. (**A**, **B**) MCF-7 cells were treated with caffeine (0.6, 1.25, 2.5, 5 mM), caffeic acid (6.25, 12.5, 25, 50 μM), chlorogenic acid (6.25, 12.5, 25, 50 μM), pyrocatechol (1.25, 2.5, 5, 10 μM), or trigonelline (12.5, 25, 50, 100 pM) for 24 h. (**A**) Whole cell lysates were immunoblotted with an anti-ERα antibody or anti-β-actin antibody. The relative expression levels of ERα are shown in the graphs. Results represent the mean ± SD of three independent experiments. *p < 0.05, **p < 0.01 significantly different from control cells. (**B**) The expression of ERα mRNA was assessed by an RT-PCR analysis. The expression of GAPDH mRNA was used as an internal control. Values are given as the mean ± SD of three independent experiments. ***p < 0.001 significantly different from control cells. (**C**) MCF-7 cells were treated with caffeine (2.5, 5 mM) in the presence of tamoxifen (2.5, 5 μM) for 24 h. The mRNA expression of c-Myc, TFF1, CTSD, GREB, and PgR was assessed by an RT-PCR analysis. The expression of GAPDH mRNA was used as an internal control. Values are given as the mean ± SD of three independent experiments. *p < 0.05, **p < 0.01, ***p < 0.001 significantly different from control cells. ^#^*p* < 0.05, ^##^*p* < 0.01, ^###^*p* < 0.001 significantly different from cells treated with 2.5 μM tamoxifen. ^§^*p* < 0.05, ^§§^*p* < 0.01, ^§§§^*p* < 0.001 significantly different from cells treated with 5 μM tamoxifen.

As shown in Figs. 1, 2, 3 and 4, caffeine contained in coffee decoction appeared to be essential for inducing cell-cycle arrest, apoptotic cell death, down-regulation of ERα expression and activation of p53. Therefore, we investigated whether caffeine alone exerts similar biological effects to coffee decoction. As shown in Fig. 9A, caffeine reduced the expression of the ERα protein, while other components in coffee decoction, such as caffeic acid, chlorogenic acid, pyrocatechol, and trigonelline, did not. We also analyzed the mRNA expression of ERα, and observed similar effects in that only caffeine down-regulated its mRNA expression (Fig. 9B). Furthermore, caffeine alone and the co-treatment with caffeine and tamoxifen inhibited the mRNA expression of the ERα target genes, c-Myc, TFF1, CTSD, GREB1, and PgR (Fig. 9C). We previously quantified the concentration of caffeine in coffee decoction and showed that about 25 mM caffeine was contained in coffee, indicating that the concentration of caffeine included in 5 v/v% coffee was around 1 mM. Therefore, it was thought that the activity of coffee decoction to reduce the expression of ERα could be due to caffeine.

**Figure 10:**
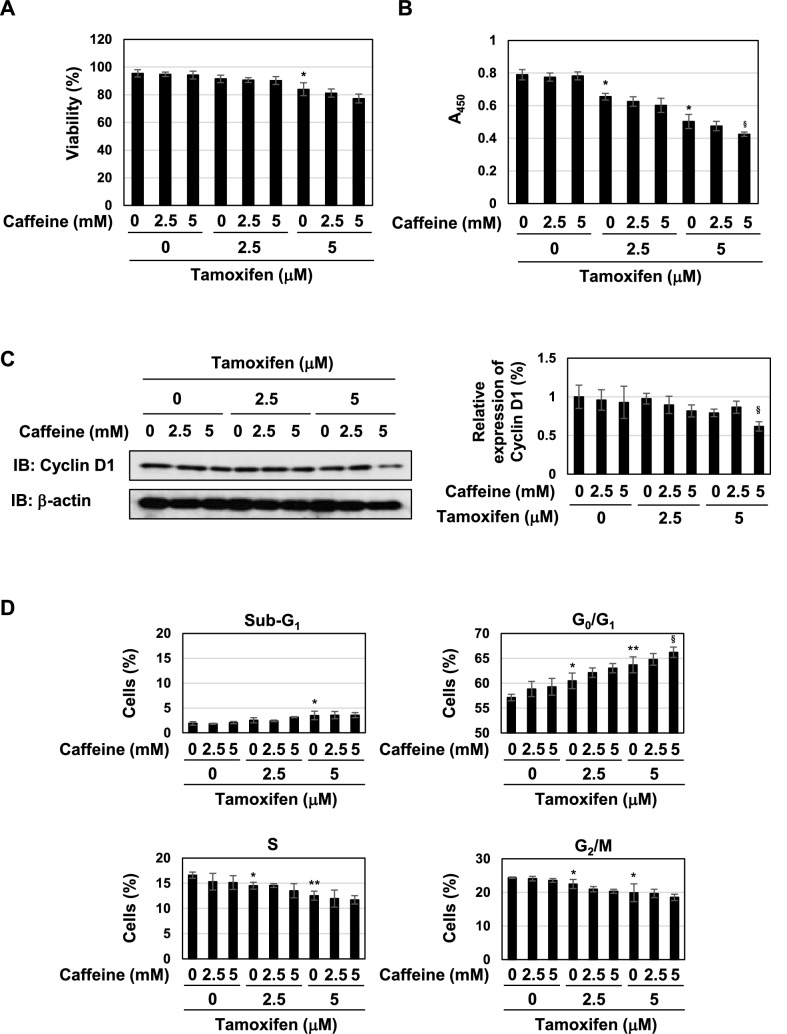

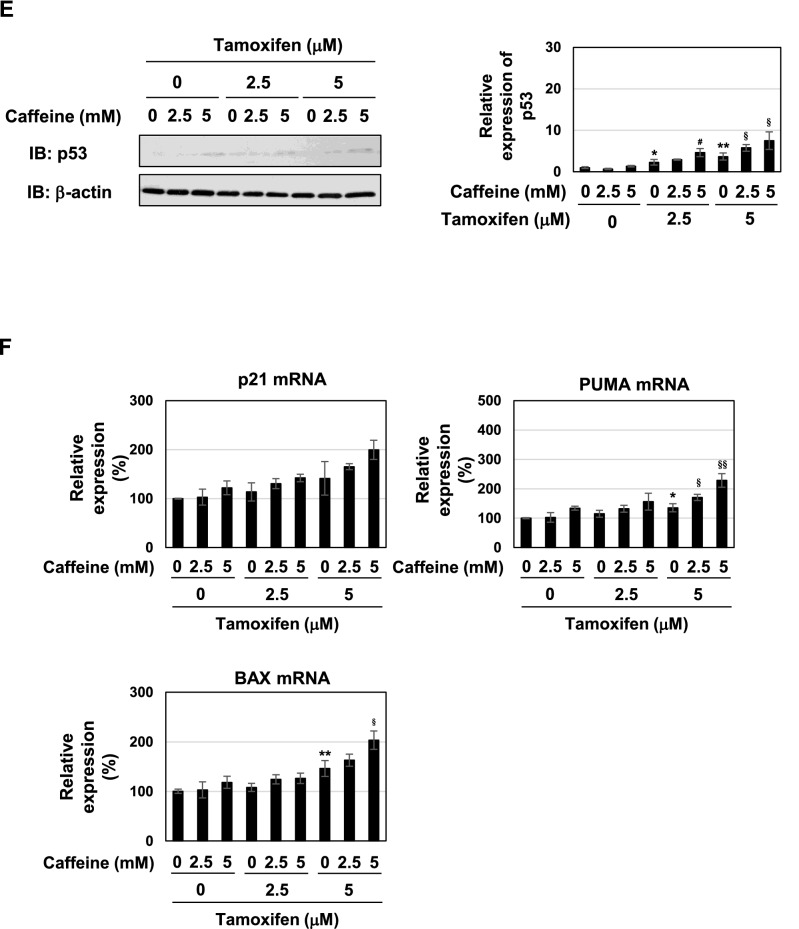

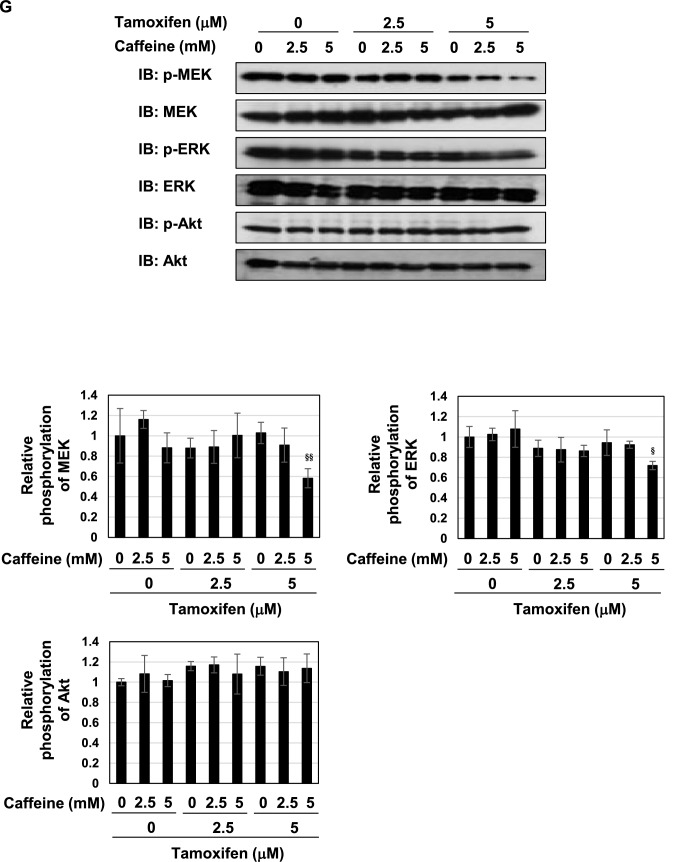
Caffeine does not enhance cell death and induce activation of p53 and inhibition of MEK, ERK and Akt in MCF-7 cells treated with tamoxifen. MCF-7 cells were treated with caffeine (2.5, 5 mM) in the presence of tamoxifen (2.5, 5 μM) for 24 h. (**A**) Cell viability was measured by the trypan blue exclusion method. Results represent the mean ± SD of three independent experiments. *p < 0.05 significantly different from control cells. (**B**) The proliferation rate was determined by BrdU incorporation assay. Results represent the mean ± SD of four independent experiments. *p < 0.05, ^§^*p* < 0.05 significantly different from control cells and cells treated with 5 μM tamoxifen, respectively. (**C**) Whole cell lysates were immunoblotted with an anti-cyclin D1 antibody or anti-β-actin antibody. The relative expression levels of cyclin D1 are shown in the graph. Results represent the mean ± SD of three independent experiments. ^§^*p* < 0.05 significantly different from cells treated with 5 μM tamoxifen. (**D**) Cells were fixed, permeabilized and treated with propidium iodide, and the cell cycle was examined using a flow cytometric analysis. The ratios of cells in the Sub-G_1_ phase, G_0_/G_1_ phase, S phase and G_2_/M phase were graphed. Data were expressed as means ± SD (n = 3). *p < 0.05, **p < 0.01 significantly different from control cells. ^§^*p* < 0.05 significantly different from cells treated with 5 μM tamoxifen. (**E**) Whole cell lysates were immunoblotted with an anti-p53 antibody or anti-β-actin antibody. The relative expression levels of p53 are shown in the graph. Results represent the mean ± SD of three independent experiments. *p < 0.05, **p < 0.01 significantly different from control cells. ^#^*p* < 0.05, ^§^*p* < 0.05 significantly different from cells treated with 2.5 μM tamoxifen and 5 μM tamoxifen, respectively. (**F**) The mRNA expression of p21, PUMA, and BAX was assessed by an RT-PCR analysis. The expression of GAPDH mRNA was used as an internal control. Values are given as the mean ± SD of three independent experiments. *p < 0.05, **p < 0.01 significantly different from control cells. ^§^*p* < 0.05, ^§§^*p* < 0.01 significantly different from cells treated with 5 μM tamoxifen, respectively. (**G**) Whole cell lysates were immunoblotted with an anti-phospho-MEK, anti-MEK, anti-phospho-ERK, anti-ERK, anti-phospho-Akt, or anti-Akt antibody. The relative phosphorylation levels of MEK, ERK and Akt are shown in the graphs. Results represent the mean ± SD of three independent experiments. ^§^*p* < 0.05, ^§§^*p* < 0.01 significantly different from cells treated with 5 μM tamoxifen.

We investigated the effects of caffeine on the viability and proliferation of MCF-7 cells. As shown in Fig. 10A–C, the treatment with caffeine alone had no effect on the viability and proliferation rate and expression level of cyclin D1 in MCF-7 cells. The co-treatment with 5 mM caffeine reduced proliferation rate and expression of cyclin D1in MCF-7 cells treated with 5 μM tamoxifen (Fig. 10B,C). We also analyzed the cell-cycle population, and found that caffeine alone did not affect cell cycle of MCF-7 cells. The co-treatment with 5 mM caffeine increased the percentage in the G_0_/G_1_ phase of cells treated with 5 μM tamoxifen (Fig. 10D). We also examined the effects of other components in coffee decoction on cell proliferation, but failed to show inhibitory effects on the proliferation of MCF-7 cells (Supplementary Fig. S3). The treatment with caffeine alone did not induce the accumulation of p53 and the expression of p53-target genes such as p21^Cip1^, PUMA, and BAX (Fig. 10E,F). The co-treatment with caffeine increased the expression of p53, PUMA mRNA and BAX mRNA, induced by the treatment with tamoxifen, however; its activity was smaller than that of coffee decoction as shown in Figs. 4A and 10E,F. In addition, caffeine alone did not inhibit activation of MEK, ERK and Akt, and the co-treatment with 5 mM caffeine and 5 μM tamoxifen reduced the phosphorylation level of MEK and ERK (Fig. 10G). These results suggest that caffeine is essential, but not sufficient for the coffee decoction-induced apoptotic cell death, full activation of p53, and inhibition of MEK, ERK and Akt.

### Components in decaffeinated coffee cooperate with caffeine and induce p53 activation and apoptosis in the presence of tamoxifen

**Figure 11:**
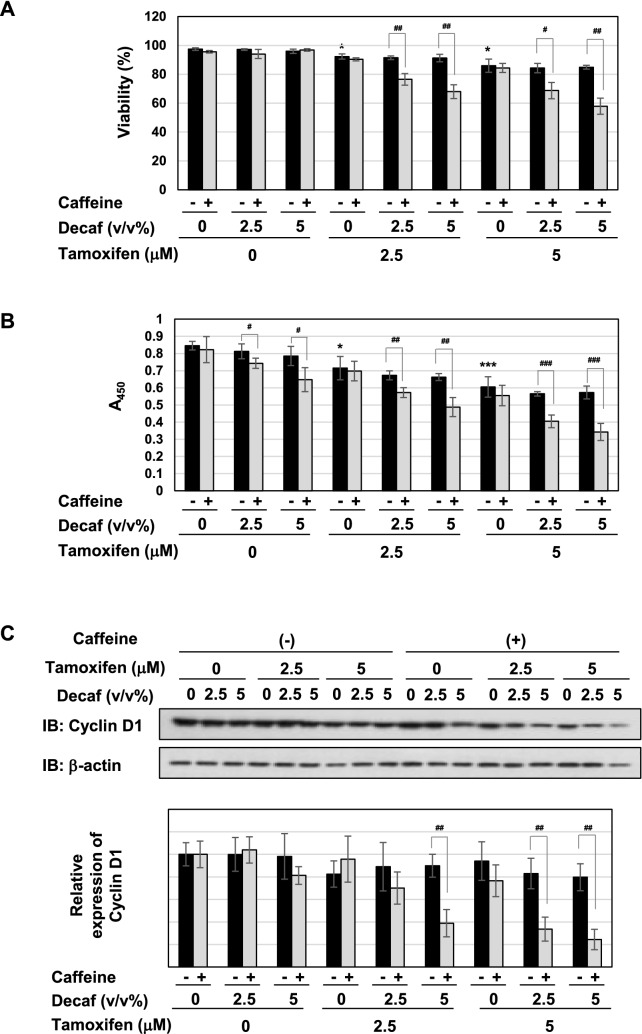

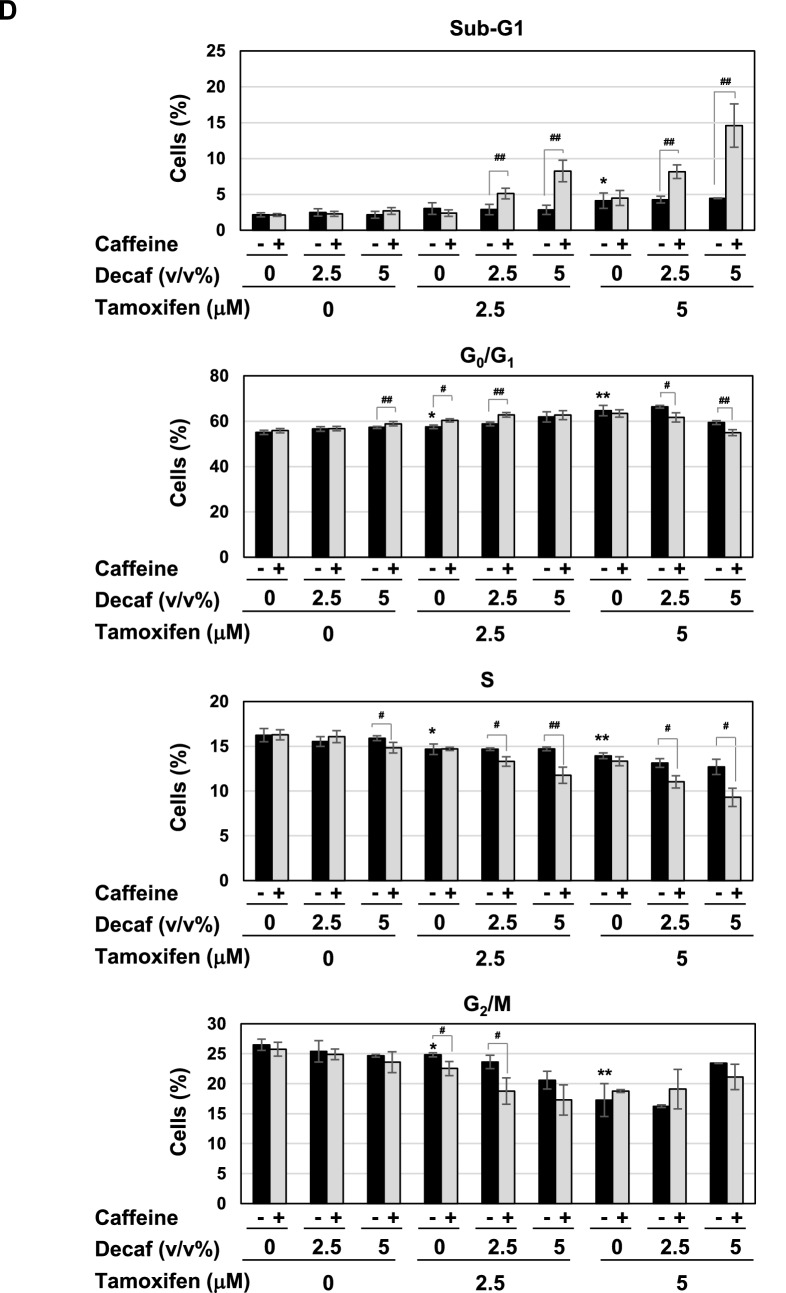

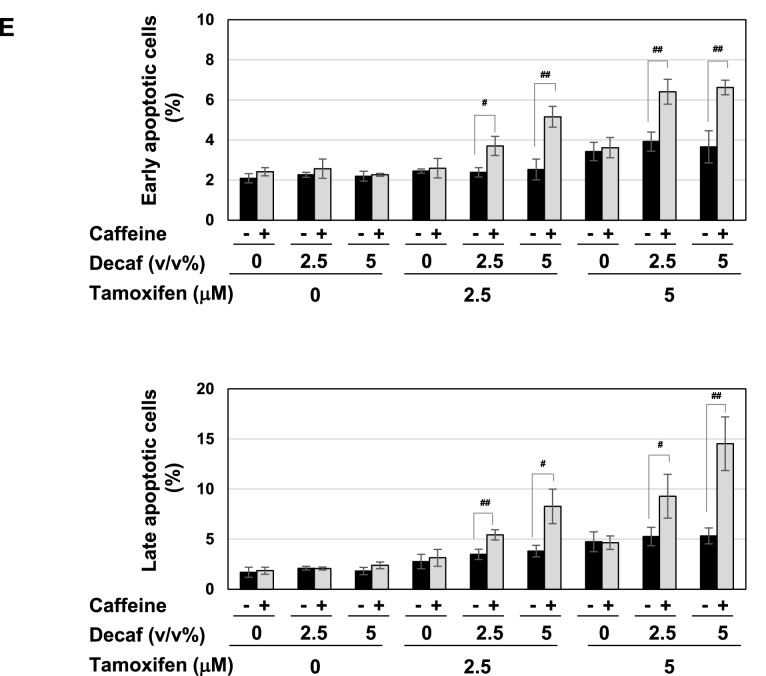
Co-treatment with decaffeinated coffee, caffeine and tamoxifen induces apoptosis in MCF-7 cells. MCF-7 cells were treated with decaffeinated coffee (2.5, 5 v/v%) with/without caffeine (2 mM) in the presence and absence of tamoxifen (2.5, 5 μM) for 24 h. (**A**) Cell viability was measured by the trypan blue exclusion method. Results represent the mean ± SD of three independent experiments. *p < 0.05 significantly different from control cells. # and ## indicates *p* < 0.05 and *p* < 0.01, respectively. (**B**) The proliferation rate was determined by BrdU incorporation assay. Results represent the mean ± SD of four independent experiments. *p < 0.05, ***p < 0.001 significantly different from control cells. #, ## and ### indicate *p* < 0.05, *p* < 0.01 and *p* < 0.001, respectively. (**C**) Whole cell lysates were immunoblotted with an anti-cyclin D1 antibody or anti-β-actin antibody. The relative expression levels of cyclin D1 are shown in the graph. Results represent the mean ± SD of three independent experiments. ## indicates *p* < 0.01. (**D**) Cells were fixed, permeabilized and treated with propidium iodide, and the cell cycle was examined using a flow cytometric analysis. The ratios of cells in the Sub-G1 phase, G_0_/G_1_ phase, S phase and G_2_/M phase were graphed. Data were expressed as means ± SD (n = 3). *p < 0.05, **p < 0.01 significantly different from control cells. # and ## indicates *p* < 0.05 and *p* < 0.01, respectively. (**E**) MCF-7 cells were treated with decaffeinated coffee (2.5, 5 v/v%) with/without caffeine (2 mM) in the presence and absence of tamoxifen (2.5, 5 μM) for 18 h. Annexin V–FITC and propidium iodide (PI) double staining was performed. The ratios of early-phase apoptotic cells and late-phase apoptotic cells were graphed. Data were expressed as means ± SD (n = 3). # and ## indicates *p* < 0.05 and *p* < 0.01, respectively.

The present results suggested the essential role of caffeine; however, unknown compounds other than caffeine were still required to induce cell-cycle arrest and apoptosis, possibly via p53. To elucidate whether decaffeinated coffee contains the required components, which cooperate with caffeine to induce p53-mediated apoptosis, we examined the effects of the combination of caffeine and decaffeinated coffee on cell-cycle arrest and apoptotic cell death. Although the combination of decaffeinated coffee decoction and caffeine had no effect on the viability of MCF-7 cells, it significantly reduced the viability of cells treated with tamoxifen (Fig. 11A). The combination of decaffeinated coffee decoction and caffeine reduced the proliferation rate of MCF-7 cells and markedly reduced the proliferation rate of MCF-7 cells treated with tamoxifen (Fig. 11B). In addition, the combination of decaffeinated coffee decoction and caffeine significantly reduced the expression of cyclin D1 in MCF-7 cells treated with tamoxifen (Fig. 11C). These results well fit with the alternation of cell cycle in MCF-7 cells treated with decaffeinated coffee, caffeine and tamoxifen as shown in Fig. 11D. Especially, the combination of decaffeinated coffee decoction and caffeine markedly increased those in the sub-G_1_ phase and significantly reduced those in S cells in the presence of tamoxifen (Fig. 11D). Furthermore, the combination of decaffeinated coffee and caffeine markedly induced apoptosis in MCF-7 cells in cooperation with tamoxifen (Fig. 11E).

**Figure 12:**
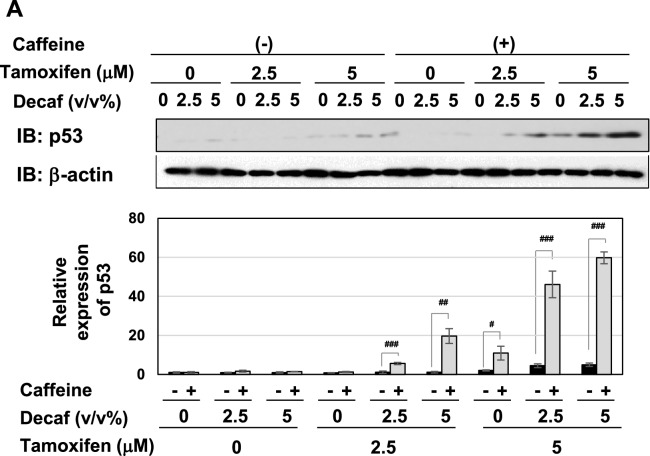

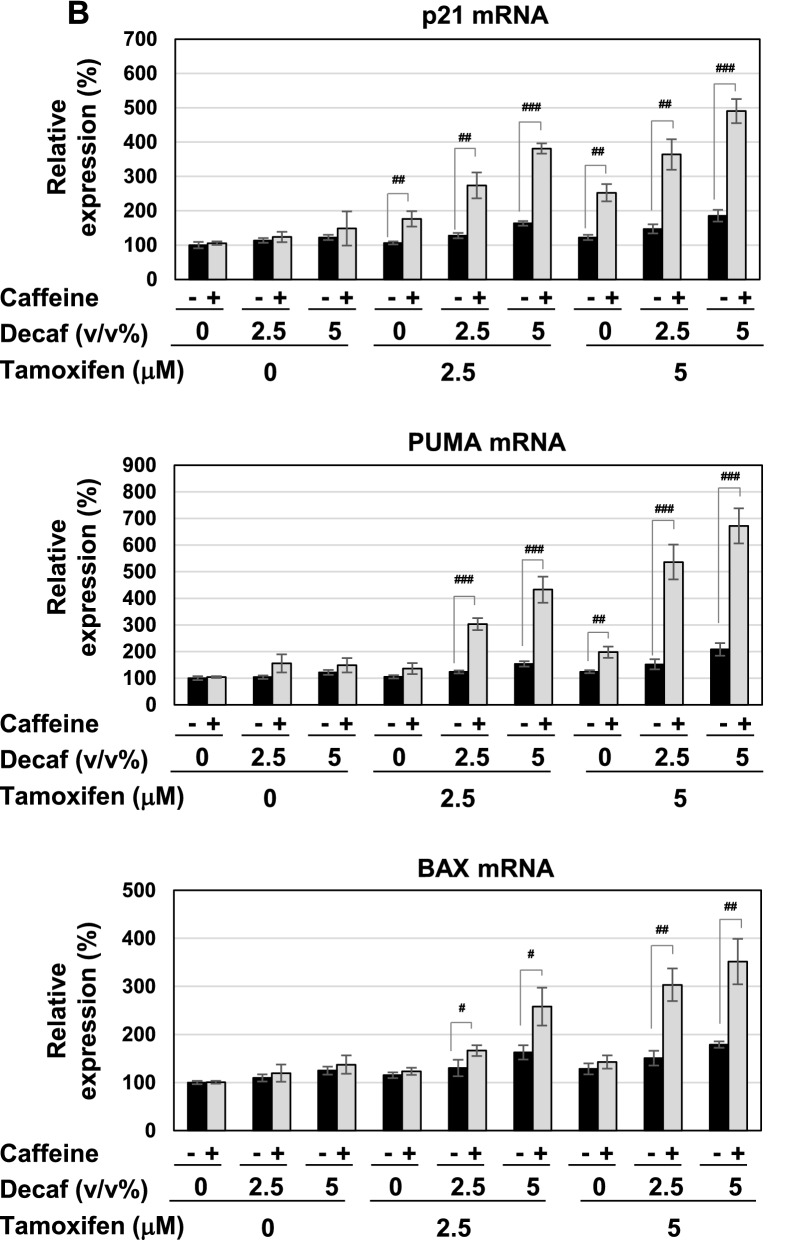

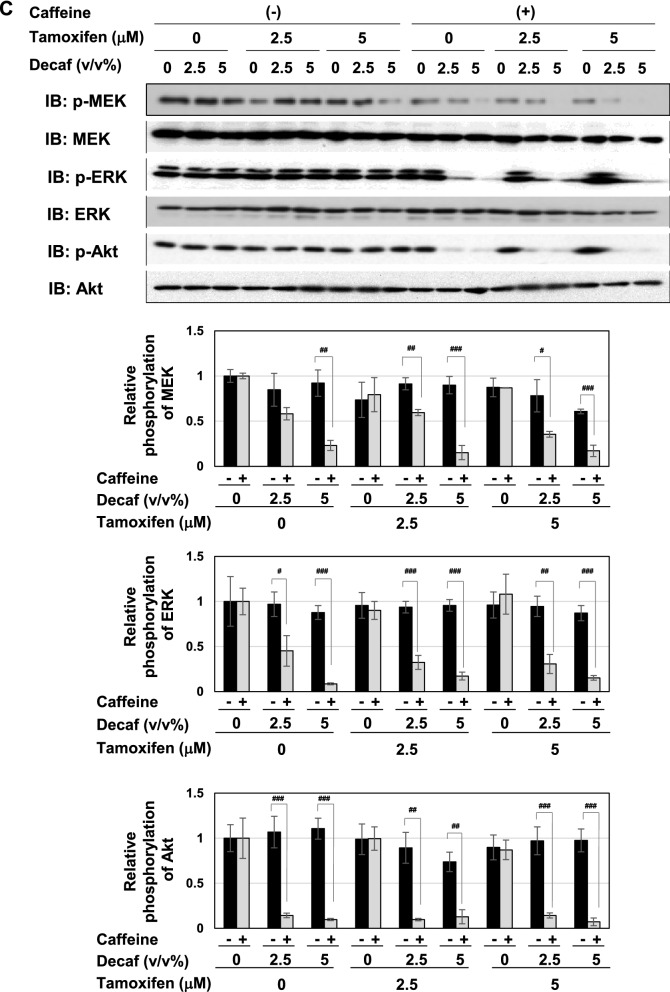
Co-treatment with decaffeinated coffee, caffeine and tamoxifen induces activation of p53 and inhibits the activation of MEK, ERK, and Akt and in MCF-7 cells. MCF-7 cells were treated with decaffeinated coffee (2.5, 5 v/v%) with/without caffeine (2 mM) in the presence and absence of tamoxifen (2.5, 5 μM) for 24 h. (**A**) Whole cell lysates were immunoblotted with an anti-p53 antibody or anti-β-actin antibody. The relative expression levels of p53 are shown in the graph. Results represent the mean ± SD of three independent experiments. #, ## and ### indicate *p* < 0.05, *p* < 0.01 and *p* < 0.001, respectively. (**B**) The mRNA expression of p21, PUMA, and BAX was assessed by an RT-PCR analysis. The expression of GAPDH mRNA was used as an internal control. Values are given as the mean ± SD of three independent experiments. #, ## and ### indicate *p* < 0.05, *p* < 0.01 and *p* < 0.001, respectively. (**C**) Whole cell lysates were immunoblotted with an anti-phospho-MEK, anti-MEK, anti-phospho-ERK, anti-ERK, anti-phospho-Akt, anti-Akt antibody, or anti-β-actin antibody. The relative phosphorylation levels of MEK, ERK, and Akt are shown in the graphs. Results represent the mean ± SD of three independent experiments. #, ## and ### indicate *p* < 0.05, *p* < 0.01 and *p* < 0.001, respectively.

Strikingly, although the combination of decaffeinated coffee decoction and caffeine failed to induce p53 accumulation, it significantly induced p53 accumulation in the presence of tamoxifen (Fig. 12A). The combination of decaffeinated coffee and tamoxifen markedly induced the mRNA expression of p21^Cip1^, PUMA, and BAX in cooperation with tamoxifen (Fig. 12B). On the other hand, the combination of decaffeinated coffee and caffeine significantly inhibited the activation of MEK, ERK and Akt regardless of the presence of tamoxifen (Fig. 12C).

**Figure 13:**
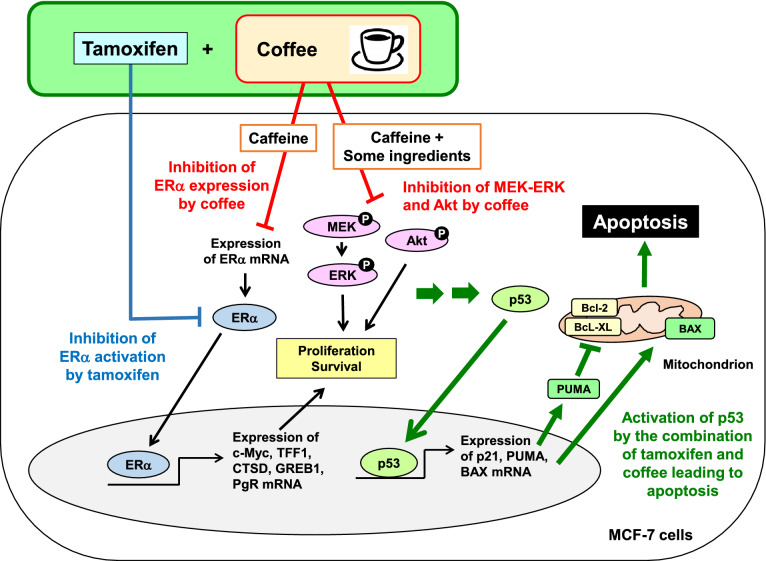
The co-treatment with coffee decoction and tamoxifen markedly induces apoptosis by activating p53 in MCF-7 cells. A major ingredient of coffee, caffeine reduces the expression of ERα mRNA, which is excessively expressed in breast cancer cells. Caffeine and some ingredients cooperatively inhibit the activation of the MEK-ERK pathway and Akt, which are critical for the proliferation of breast cancer cells. The co-treatment with coffee and tamoxifen cooperatively induce apoptosis by strongly activating p53 in MCF-7 cells.

Collectively, the present results demonstrated that coffee decoction cooperates with tamoxifen to induce apoptosis through p53 activation in ERα-positive breast cancer cells harboring wild type p53. Coffee decoction inhibits the expression of ERα, and this action is due to caffeine contained in coffee. Coffee decoction also inhibits activation of MEK-ERK pathway and ERK, and this inhibition is mediated by caffeine and other unknown compounds in coffee. However, it is still unknown how the downregulation of ERα and inhibition of MEK, ERK and Akt by coffee are related to the enhancement of anti-tumor activity of tamoxifen (Fig. 13).

## Discussion

Tamoxifen resistance is a critical issue in therapeutic strategies for breast cancer. The loss of ERα expression and its function involve a switch from ERα-positive to ERα-negative tumors^14,15^. In addition, various mechanisms underlying resistance to tamoxifen have been proposed such as the appearance of constitutive active mutant of ERα, expression of HER2, and activation of the Ras/MEK/ERK and PI3K/Akt pathways^16–22^.

In the present study, caffeine in coffee decoction was sufficient to reduce ERα expression (Figs. 1, 9). As shown in Figs. 3 and 9C, coffee decoction and caffeine significantly reduced the expression of ERα-target genes such as c-Myc, TFF1, CTSD, GREB, and PgR as well as tamoxifen. Whereas the treatment with coffee decoction alone or tamoxifen alone reduced the proliferation of MCF-7 cells, the treatment with caffeine alone had no effect on the proliferation of MCF-7 cells (Figs. 1B, 10B). Although we need to examine the effects of coffee, caffeine and tamoxifen on the protein expression levels of c-Myc, TFF1, CTSD, GREB, and PgR, it is thought the possibility that the downregulation of ERα-target genes is insufficient to inhibit the proliferation of MCF-7 cells.

In this study, the combined treatment with coffee decoction and tamoxifen cooperatively induced apoptosis in MCF-7 cells (Fig. 1). The downregulation of ERα by coffee decoction seems to be linked to the occurrence of tamoxifen resistance. Coffee decoction did not exert cytotoxicity and tamoxifen decreased the viability of MCF-7 cells to 70% (Fig. 1). Since the expression of ERα was reduced but still detected when treated with coffee decoction with/without tamoxifen in MCF-7 cells (Fig. 2C), it was thought that tamoxifen was still able to antagonize ERα. As a result, coffee decoction and tamoxifen inhibit the activity of ERα by reducing the expression of ERα mRNA and antagonizing ERα, respectively. Previously, Rosendahl et al*.* showed that the consumption of coffee improved the curative effect of the ERα-positive breast cancer with tamoxifen by an epidemiologic study^32^. It is suggested that the expression level of ERα in breast tumor tissues would also decreased by consumption of coffee and this situation can become the condition that tamoxifen can effectively antagonize ERα. Interestingly, the co-treatment with coffee decoction and tamoxifen did not decrease the viability of ERα-positive T47D cells, in which p53 is mutated (Fig. 5A,C). These data suggested that the improvement of the curative effect of tamoxifen by consumption of coffee greatly depended on the status of p53 in the breast cancer patients.

The stability of p53 is regulated by Mdm2, a ubiquitin ligase (E3)^47^. Previous studies reported that Akt enhanced the ubiquitin ligase activity of Mdm2 by phosphorylating Ser166 and Ser186, resulting in reductions in the p53 protein and the promotion of cell survival and proliferation^48,49^. Since Mdm2 is a p53 target gene, the activation of p53 also results in a negative feedback loop against p53. ERK signaling has been shown to contribute to the expression of the Mdm2 protein through the transportation of Mdm2 mRNA to the cytosol in T47D cells^50^. The treatment with coffee alone inhibited the activation of MEK, ERK and Akt (Fig. 6) and the inhibitors, U0126 and LY294002 cooperated with tamoxifen and induced apoptosis through p53 activation in MCF-7 cells (Figs. 7, 8), suggesting the possibility that coffee-induced activation of p53 is due to regulating Mdm2 through inhibition of MEK-ERK pathway and Akt. However, the treatment with coffee alone failed to induce apoptotic cell death in MCF-7 cells (Fig. 6). Since MEK-ERK and Akt are major signaling molecules which promote various biological process^51^, the effects obtained using the inhibitors are not necessarily equal to the effects by coffee-induced inhibition of MEK-ERK and Akt. It is necessary to understand the precise mechanisms how coffee decoction enhances tamoxifen anti-apoptotic activity through inhibition of MEK-ERK and Akt by analyzing the roles of downstream molecules of these signaling pathways. Although the underlying mechanisms have not yet been elucidated in detail, the co-treatment with coffee and tamoxifen appeared to activate p53, leading to apoptotic cell death through the expression of Bax and Puma which trigger for the release of cytochrome C from mitochondria^38,39^. To understand the detailed mechanism, it will be important to investigate whether the combination of coffee decoction and tamoxifen can induce apoptosis in various tamoxifen-resistant cell lines expressing active ERα mutant or HER2.

Coffee contains a number of compounds, and the roasting procedure causes chemical reactions that generate numerous products, such as pyrocatechol and various melanoidins^30,52^. Consistent with a previous study^53^, we observed that trigonelline increased the expression level of ERα and enhanced the proliferation of MCF-7 cells. On the other hand, other major coffee components, such as caffeic acid, chlorogenic acid, and pyrocatechol, had no effect on the expression of ERα or the proliferation of MCF-7 cells with/without tamoxifen (Fig. 9, Supplementary Fig. S3). Unlike a single chemical pharmaceutical which target specific signaling molecule, the activity of a mixture such as coffee decoction is different from the activity of each compound, and usually stronger than the activity of single compounds^54^. It is normally expected that the concentration, biotransformation and metabolism of compounds, as well as the matrix effect, affect the activity of a mixture. Therefore, it might be difficult to provide a clear picture explaining the activity of each component in coffee and it is important to assess the activity of coffee as a mixture.

In the present study, we focused on the effects of coffee decoction on the proliferation of and apoptosis in ERα-positive breast cancer cells. In many tumors, tumor suppressor genes, such as p53, Rb, and p16^Ink4a^, are genetically inactivated by deletions, mutations, or epigenetic silencing^55^. However, in the many case of ERα-positive breast cancer, the p53 gene is intact, and a stimulation with estrogen suppresses the transcriptional activity of p53^56^. Therefore, coffee decoction may contribute to the activation of p53 by inhibiting the transcriptional activity of ERα.

The present results indicated that coffee enhanced the efficacy of tamoxifen against breast cancer, and caffeine appears to be a critical compound for these effects. However, the cooperation of caffeine and tamoxifen was not sufficient to induce p53-mediated apoptotic cell death, and unknown compound(s) contained in coffee appear to be required (Fig. 13). To clarify the mechanisms by which coffee decoction enhances the therapeutic efficiency of tamoxifen against breast cancer in more detail, the identification of unknown compound(s) in coffee decoction will be of importance. It is also important to validate the anti-tumor activity of coffee and tamoxifen in vivo. In the future, it will be necessary to examine whether drinking coffee can enhance the anti-tumor activity by the administration of tamoxifen in nude mice transplanted with MCF-7 cells.

## Methods

### Preparation of coffee decoction

Roasted coffee powder and roasted decaffeinated coffee powder (Columbia Arabica) were purchased from Starbucks Coffee Japan (Tokyo, Japan). Eight grams of each coffee powder was poured with 140 mL of 95 °C hot water through a paper filter (Mellita, Minden, Germany) and stored at − 20 °C until used, as described previously^30^.

### Reagents and antibodies

Tamoxifen was purchased from Nacalai Tesque (Tokyo, Japan). Caffeine, caffeic acid, chlorogenic acid, pyrocatechol, and trigonelline were purchased from Sigma-Aldrich (St. Louis, MO). LY294002 and U0126 were purchased from Tocris Bioscience (Bristol, UK). Anti-ERα, anti-phospho-MEK1/2 (S217/221), anti-MEK1/2, anti-phospho-ERK1/2 (T202/Y204), anti-ERK1/2, anti-phospho-Akt (S473), anti-Akt, and anti-cleaved caspase-3 antibodies were purchased from Cell Signaling Technology (Danvers, MA, USA). Anti-p53, anti-cyclin D1, and anti-β-actin antibodies were purchased from Santa Cruz Biotechnology, Inc. (Santa Cruz, CA, USA). Peroxidase-conjugated rabbit anti-mouse, goat anti-rabbit, and swine anti-goat secondary antibodies were from Dako (Glostrup, Denmark).

### Cell culture

The human breast cancer cell line MCF-7 and osteosarcoma cell line U2OS were obtained from the American Tissue Culture Collection (Manassas, VA, USA). The human breast adenocarcinoma cell line MDA-MB-231 and colon cancer cell line HCT116 were purchased from JCRB Cell Bank (Japanese Collection of Research Bioresources Cell Bank) (Osaka, Japan). The human breast carcinoma T47D cells and murine embryonic fibroblasts (MEF) were purchased from Cell Biolabs, Inc. (San Diego, CA, USA) and LONZA (Walkersville, MD, USA), respectively. MCF-7 cells were cultured in Dulbecco’s modified Eagle’s medium (DMEM) (Nacalai Tesque, Tokyo, Japan) supplemented with 5% fetal bovine serum (FBS) (BioWest, Nuaillé, France), 100 units/mL penicillin (Nacalai Tesque), and 100 μg/mL streptomycin (Nacalai Tesque). Other cells were cultured in DMEM supplemented with 10% FBS, 100 units/mL penicillin, and 100 μg/mL streptomycin.

### Measurement of cell viability

MCF-7 cells, T47D cell, MDA-MB-231 cells, MEF, HCT116 cells and U2OS (5 × 10^5^ cells) were seeded in a 6-well plate and allowed to attach for 24 h. Cells were treated with combination of coffee decoction or decaffeinated coffee decoction (1.25, 2.5, 5, 10, 20 v/v%) with/without tamoxifen (0.6, 1.25, 2.5, 5, 10 μM) and incubated for 24 h. MCF-7 cells were treated with combination of caffeine (2.5, 5 mM), U0126 (10, 20 μM) or LY294002 (20, 30 μM) and tamoxifen (2.5, 5 μM) for 24 h. The trypan blue dye exclusion test was used to determine cell viability as described previously^30^. To evaluate whether the activity of coffee ad tamoxifen is synergistic or additive, combination index (CI) was calculated as previously reported^32^ and showed CI value in Table 1.

### BrdU cell proliferation assay

MCF-7 cells were seeded in a 96-well plate with the amounts of 2 × 10^4^ cells/well and allowed to attach for 24 h. Then, the cells were treated with decoction of coffee or decaffeinated coffee (2.5, 5 v/v%), caffeine (2.5, 5 mM), U0126 (10, 20 µM) or LY294002 (20, 30 μM) with/without tamoxifen (2.5, 5 μM) for 24 h. The proliferation rate was determined using the BrdU labeling and detection ELISA kit (Abcam, Cambridge, United Kingdom).

### Annexin-FITC/propidium iodide double staining

MCF-7 cells were seeded in a 6-well plate with the amounts of 5 × 10^5^ cells/well and allowed to attach for 24 h. Then, cells were treated with decoction of coffee or decaffeinated coffee (2.5, 5 v/v%), U0126 (10, 20 μM) or LY294002 (20, 30 μM) with/without tamoxifen for 18 h. Apoptosis assessment was conducted using flow cytometry by Annexin V–FITC and propidium iodide (PI) double staining using Annexin V-FITC Apoptosis Detection Kit (Nacalai tesque). Cells stained with only Annexin V-FITC demonstrate early-phase apoptotic cells and cells stained with both PI and Annexin V-FITC demonstrate late-stage apoptosis, respectively. The ratio of early apoptotic cells and late apoptotic cells were determined using flow cytometer BD LSR II (BD Bioscience, Franklin Lakes, NJ, USA).

### Morphological assessment

MCF-7 cells were seeded in a 6-well plate with the amounts of 2.0 × 10^5^ cells/well and allowed to attach for 24 h. Then, the cells were treated with and treated with coffee or decaffeinated coffee (2.5, 5 (v/v%) and tamoxifen (2.5, 5 μM) for 24 h. Morphological changes of MCF-7 cells were viewed using BZ-X800 Fluorescence Microscope (KEYENCE, Frankfurt, Germany). Cells were photographed through a brightfield microscope under 40 × magnification.

### Immunoblotting

MCF-7 cells, T47D cells, MDA-MB-231 cells, MEF, HCT116 cells, and U2OS cells (5 × 10^6^ cells) were seeded in a 60 mm dish and allowed to attach for 24 h. Cells were treated with combination of coffee decoction (2.5, 5 v/v%) or decaffeinated coffee decoction (2.5, 5 v/v%), U0126 (10, 20, 30 μM), LY294002 (10, 20, 30 μM) or caffeine (2.5, 5 mM) with/without tamoxifen (2.5, 5 μM) and incubated for 24 h. MCF-7 cells were treated with caffeine (0.6, 1.25, 2.5, 5 mM), caffeic acid (6.25, 12.5, 25, 50 μM), chlorogenic acid (6.25, 12.5, 25, 50 μM), pyrocatechol (1.25, 2.5, 5, 10 μM), or trigonelline (12.5, 25, 50, 100 pM) for 24 h. Cells were lysed in NP-40 lysis buffer (50 mM Tris–HCl pH 8.0, 120 mM NaCl, 1 mM EDTA, 0.5% Nonidet P-40, 20 mM NaF, 0.2 mM Na_3_VO_4_, 2 μg/mL aprotinin, and 2 μg/mL leupeptin). After the Bradford protein assay, equivalent amounts of proteins were denatured with Laemmli buffer and resolved by SDS-PAGE, and immunoblotting was then performed, as described previously^30^. The intensity of each band was quantified by ImageJ software.

### Reverse transcription-polymerase chain reaction (RT-PCR)

MCF-7 cells (5 × 10^6^ cells) were seeded in a 60 mm dish and allowed to attach for 24 h. Cells were treated with combination of coffee decoction (2.5, 5 v/v%) or decaffeinated coffee decoction (2.5, 5 v/v%), U0126 (10, 20 μM), LY294002 (20, 30 μM) or caffeine 2.5, 5 mM) with/without tamoxifen (2.5, 5 μM) and incubated for 24 h. MCF-7 cells were treated with caffeine (0.6, 1.25, 2.5, 5 mM), caffeic acid (6.25, 12.5, 25, 50 μM), chlorogenic acid (6.25, 12.5, 25, 50 μM), pyrocatechol (1.25, 2.5, 5, 10 μM), or trigonelline (12.5, 25, 50, 100 pM) for 24 h. Total RNA was decoctioned using Sepazol (Nacalai Tesque). The reverse transcription reaction and quantitative real-time PCR were performed as described previously^30^. PCR primer sequences were shown in Table 2.

**Table 2:** PCR primer sequences.

Era	5′-AGCACCCTGAAGTCTCTGGA-3′ (upstream)
	5′-GATGTGGGAGAGGATGAGGA-3′ (downstream)
c-Myc	5′-CCCTCCACTCGGAAGGACTA-3′ (upstream)
	5′-GCTGGTGCATTTTCGGTTGT-3′ (downstream)
TFF1	5′-CCCAGTGTGCAAATAAGGGC-3′ (upstream)
	5′-TGGAGGGACGTCGATGGTAT-3′ (downstream)
CTSD	5′-TTCATCGGCCGCTACTACAC-3′ (upstream)
	5′-CTGCTCTGGGACTCTCCTCT-3′ (downstream)
GREB1	5′-GCACATCTTGTACTTTGATGCCC-3′ (upstream)
	5′-TGTCCTCAGCTGCAGTTTGTT-3′ (downstream)
PgR	5′-CGGTCCAGCCACATTCAACA-3′ (upstream)
	5′-GTCATGACGACTGGACTCCC-3′ (downstream)
p21	5′-CACCACTGGAGGGTGACTTC-3′ (upstream)
	5′-CGTGGGAAGGTAGAGCTTGG-3′ (downstream)
PUMA	5′-AAATTTGGCATGGGGTCTGC-3′ (upstream)
	5′-CCTCTTGTCTCACAAATCTGGC-3′ (downstream)
BAX	5′-CCAGCTCTTTAATGCCCGTTC-3′ (upstream)
	5′-CAGTCGCTTCAGTGACTCGG-3′ (downstream)
BAX	5′-ACTCCACTCACGGCAAATTC-3′ (upstream)
	5′-CCTTCCACAATGCCAAAGTT-3′ (downstream)

### Cell-cycle analysis

MCF-7 cells (5 × 10^6^ cells) were seeded in a 60 mm dish and allowed to attach for 24 h. Cells were treated with combination of coffee decoction (2.5, 5 v/v%), decaffeinated coffee decoction (2.5, 5 v/v%), U0126 (10, 20, 30 μM), LY294002 (10, 20, 30 μM) or caffeine 2.5, 5 mM) with/without tamoxifen (2.5, 5 μM) and incubated for 24 h. After cells had been fixed with 70% (v/v) cold ethanol at − 20 °C overnight, they were treated with 10 μg/ml RNase A (Wako, Tokyo, Japan) and stained with 100 μg/ml propidium iodide (Sigma). The cell cycle was analyzed by measuring the content of DNA, as described previously^57^.

### Statistical analysis

Data are indicated as the mean ± SD. All experiments were repeated at least three times. The significance of differences between mean values for the treatment groups were calculated by the Student’s *t*-test and a one- or two-way analysis of variance (ANOVA) followed by Tukey’s test. A *p*-value < 0.05 was considered to be significant.

## Supplementary information

Supplementary Figures.

## Abbreviations

CTSD: Cathepsin D
Erα: Estrogen receptor α
ERE: Estrogen response elements
FBS: Fetal bovine serum
GREB1: Growth regulation by estrogen in breast cancer 1
PgR: Progesterone receptor
TFF1: Trefoil factor 1
