# Supplementary material for: Coffee decoction enhances tamoxifen proapoptotic activity on MCF-7 cells
Source: Sci Rep. 2020 Nov 11;10:19588. doi: 10.1038/s41598-020-76445-z (PMC7659352; doi:10.1038/s41598-020-76445-z)

Supplemental information

Coffee decoction enhances tamoxifen proapoptotic activity on MCF-7 cells

Megumi Funakoshi-Tago, Kenji Tago, Chin Li, Shingo Hokimoto and Hiroomi Tamura

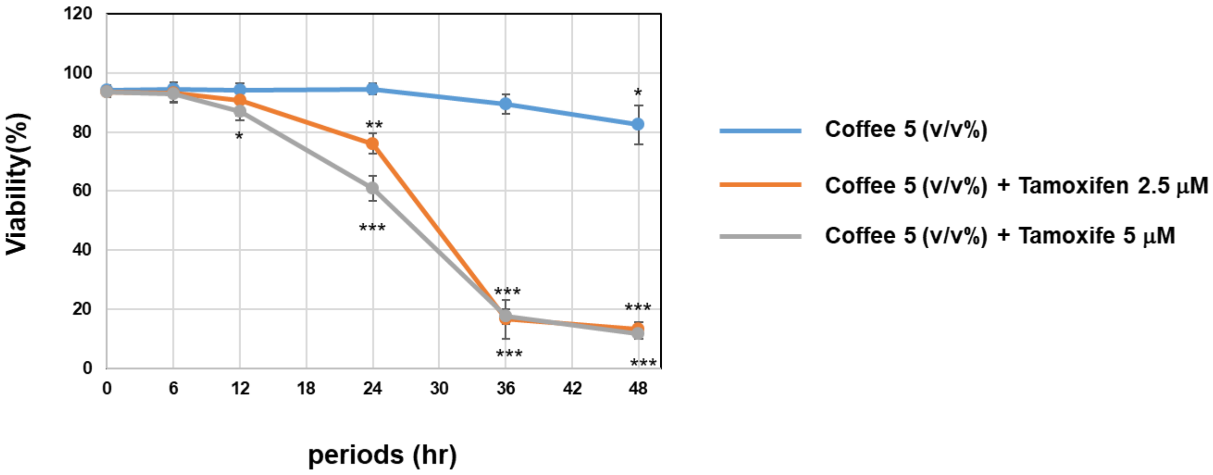

Supplemental Figure S1

The combination of coffee decoction and tamoxifen significantly induces cell death at 24 hr in MCF-7 cells.

MCF-7 cells (5×10^5 cells) were treated with coffee decoction (5v/v%) with/without tamoxifen (2, 5 µM, 5 µM) for 6, 12, 24, 36 and 48 hr. Cell viability was determined by the trypan blue exclusion method. Results represent the mean ± SD of three independent experiments. *p<0.05, **p<0.01, ***p<0.01, significantly different from control cells.

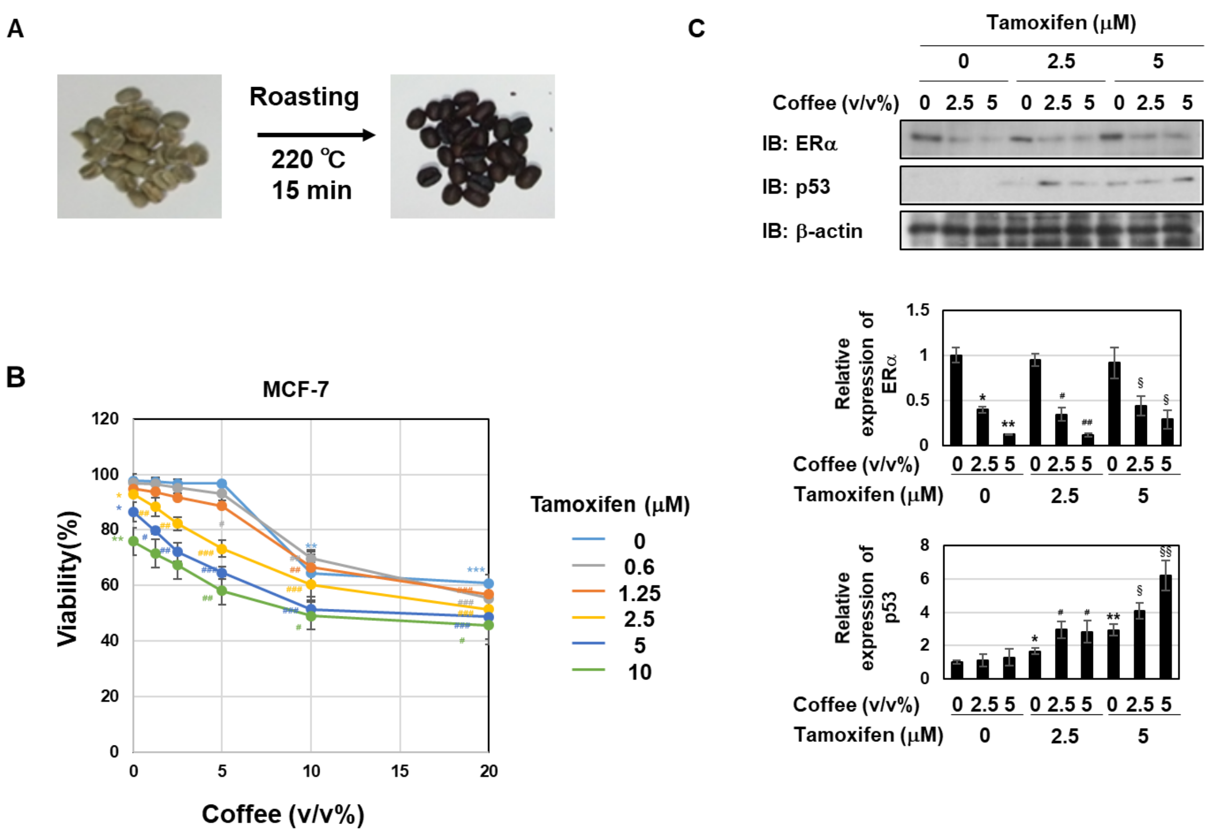

Supplemental Figure S2

The decoction of roasted coffee beans induces cell death, downregulation of ERα and expression of p53 in MCF-7 cells.

(A) Green coffee beans (Columbia Arabica) were obtained from Yumekobo (Tokyo, Japan) and roasted at 220 degrees for 15 min using Coffee Roaster MR-101 (DAINICHI, Niigata, Japan) and their decoctions were prepared as described in Methods. (B) MCF-7 cells (5×10^5 cells) were treated with coffee decoction (1.25, 2.5, 5, 10, 20 % (v/v)) with/without tamoxifen (0.6, 1.25, 2.5, 5, 10 μM) for 24 hr. Cell viability was determined by the trypan blue exclusion method. Results represent the mean ± SD of three independent experiments. *$p<0.05$, **$p<0.01$, ***$p<0.01$ significantly different from control cells. #$p<0.05$, ##$p<0.01$, ###$p<0.01$ significantly different from cells treated with tamoxifen. (C) MCF-7 cells (5×10^6 cells) were treated with coffee decoction (2.5, 5

(v/v)) in the absence or presence of tamoxifen (2.5, 5 μM) for 24 hr. Cell lysates were immunoblotted with an anti-ERα, anti-p53, or anti-β-actin antibody. The intensity of each band was quantified by ImageJ software and the relative expression levels of ERα and p53 were shown in graphs. Results represent the mean ± SD of three independent experiments. *p<0.05, **p<0.01 significantly different from control cells. #p<0.05, ##p<0.01 significantly different from cells treated with 2.5 μM tamoxifen. §p<0.05, §§p<0.05 significantly different from cells treated with 5 μM tamoxifen.

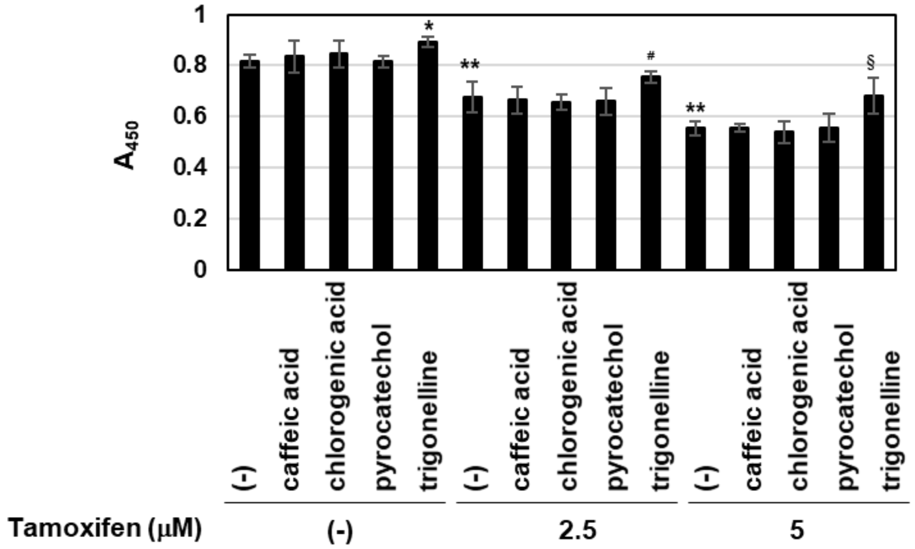

Supplemental Figure S3

Caffeic acid, chlorogenic acid, pyrocatechol, and trigonelline fail to inhibit the proliferation rate of MCF-7 cells in the absence and presence of tamoxifen

MCF-7 cells (2×10^4 cells) were treated with caffeic acid (50 μM), chlorogenic acid (50 μM), pyrocatechol (10 μM), or trigonelline (100 pM) in the absence or presence of tamoxifen (2.5, 5 μM) for 24 hr. The proliferation rate was determined using the BrdU labeling and detection ELISA kit (abcam). Results represent the mean ± SD of three independent experiments. *p<0.05, **p<0.01 significantly different from control cells, # and § indicate p<0.05 significantly different from cells treated with 2.5 μM tamoxifen and 5 μM tamoxifen, respectively.

Supplemental Figure S4
Photographs of the full-length blots utilized in Figures

Fig. 1C

IB: Cyclin D1 (Coffee)

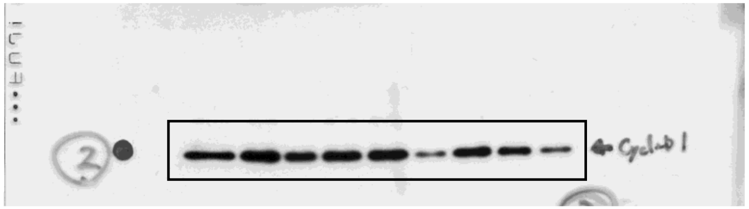

IB: Cyclin D1 (Decaf)

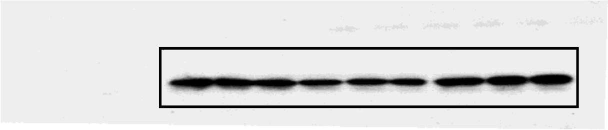

IB: β-actin (Coffee) **IB: β-actin (Decaf)**

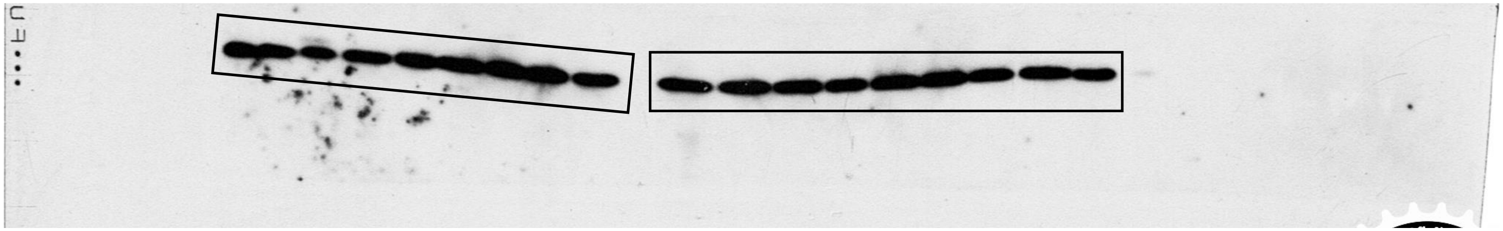

Fig. 2A

IB: ERα

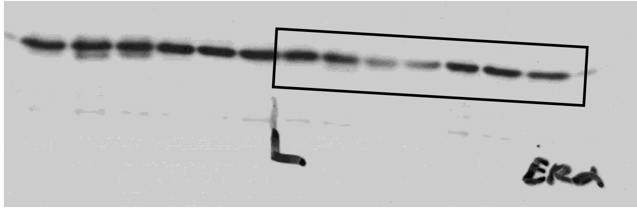

IB: β-actin

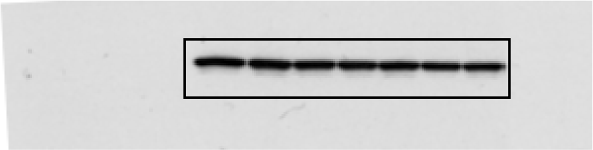

Fig. 2C

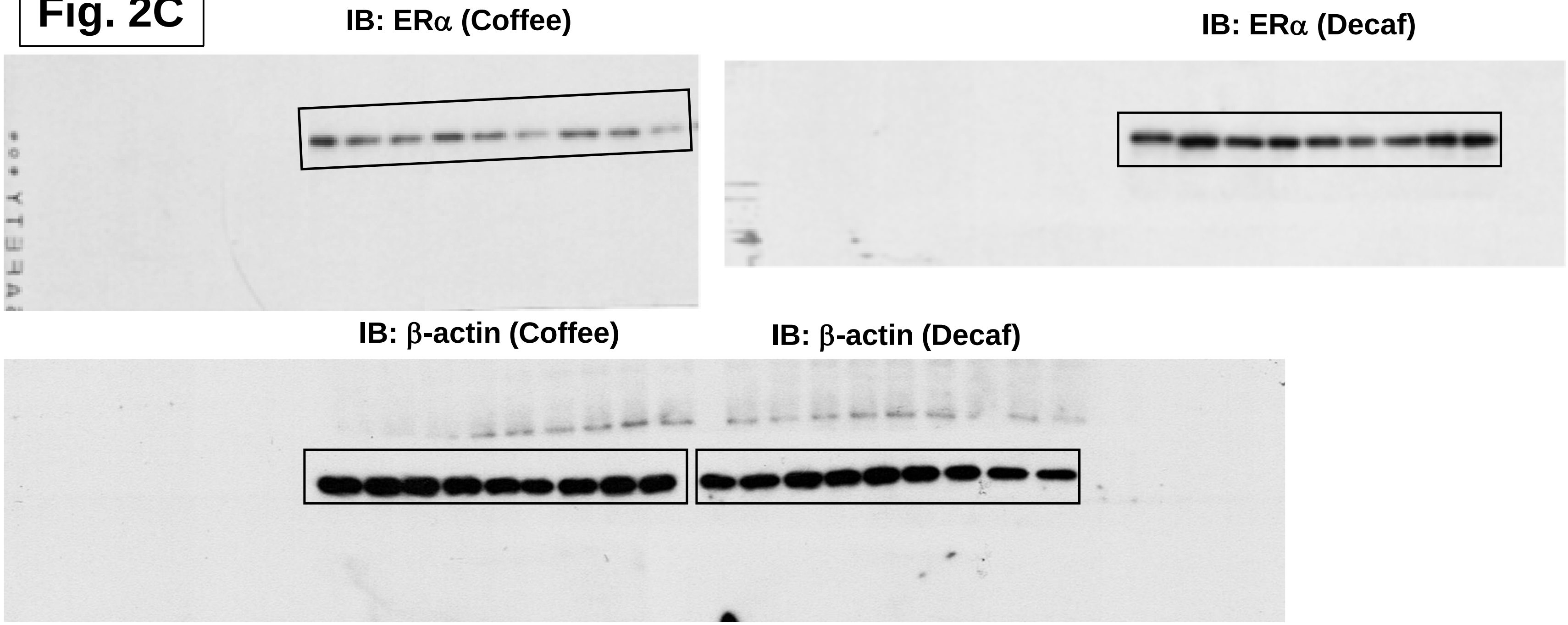
IB: ERα (Coffee)
IB: ERα (Decaf)
IB: β-actin (Coffee)
IB: β-actin (Decaf)

Fig. 4A

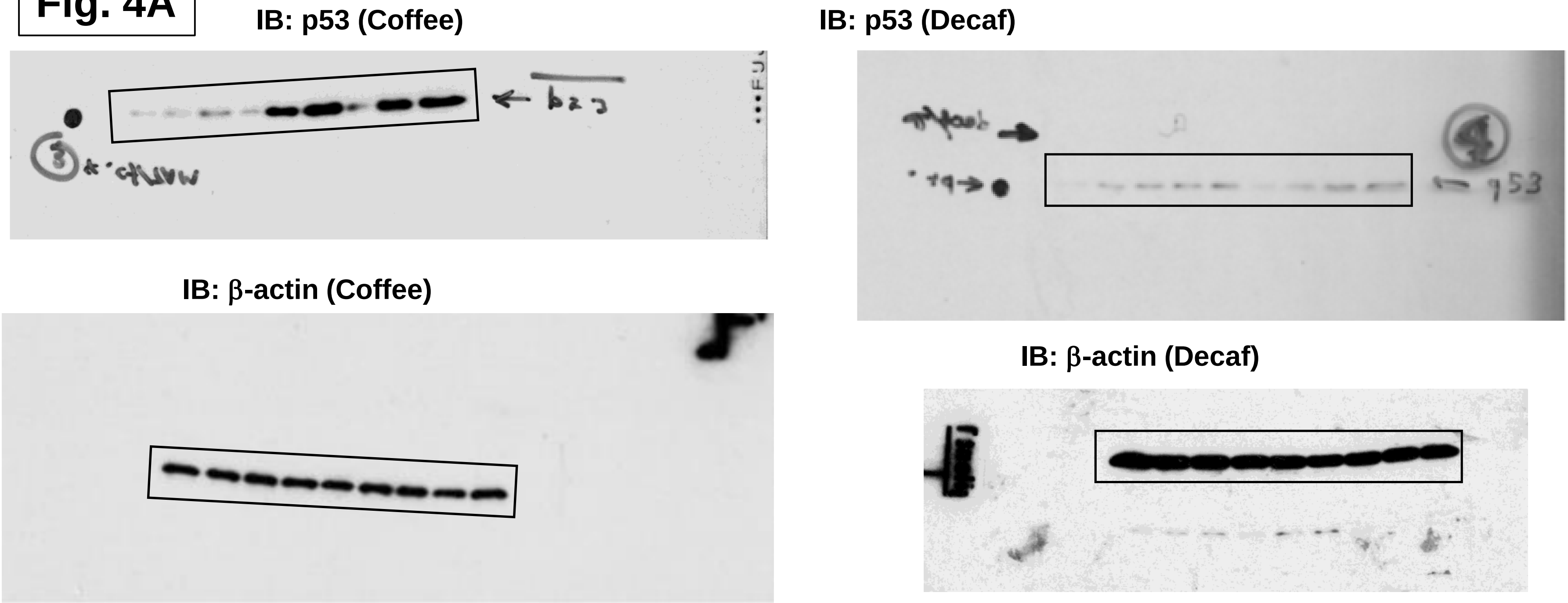
IB: p53 (Coffee)
IB: p53 (Decaf)
IB: β-actin (Coffee)
IB: β-actin (Decaf)

Fig. 5C

IB: ERα

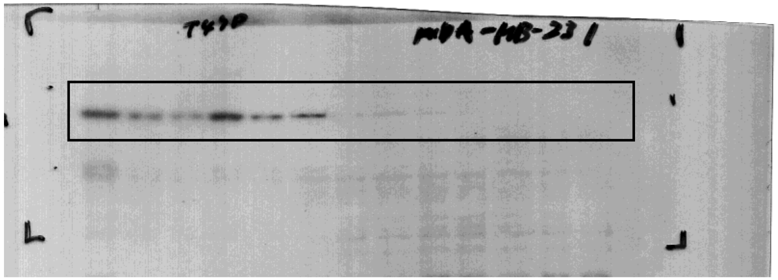

IB: p53

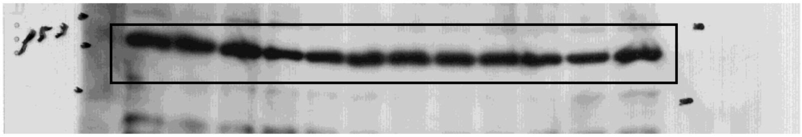

IB: β-actin

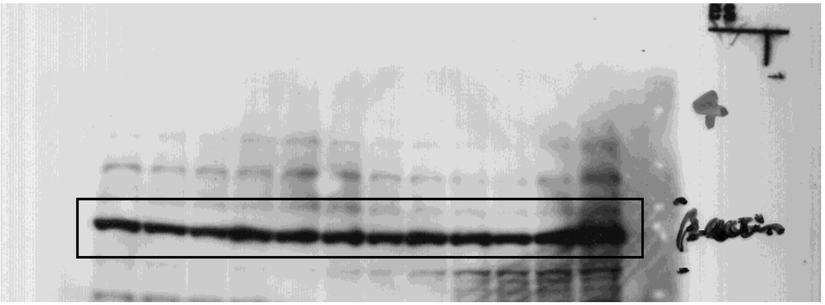

Fig. 5D

IB: p53 (MEF)

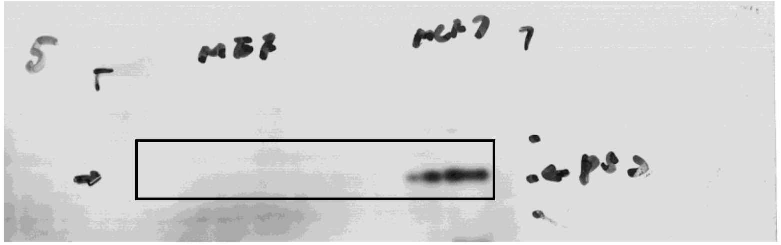

IB: β-actin (MEF)

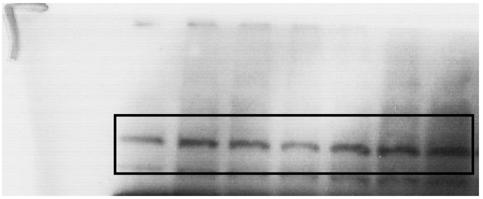

IB: p53 (HCT116, U2OS)

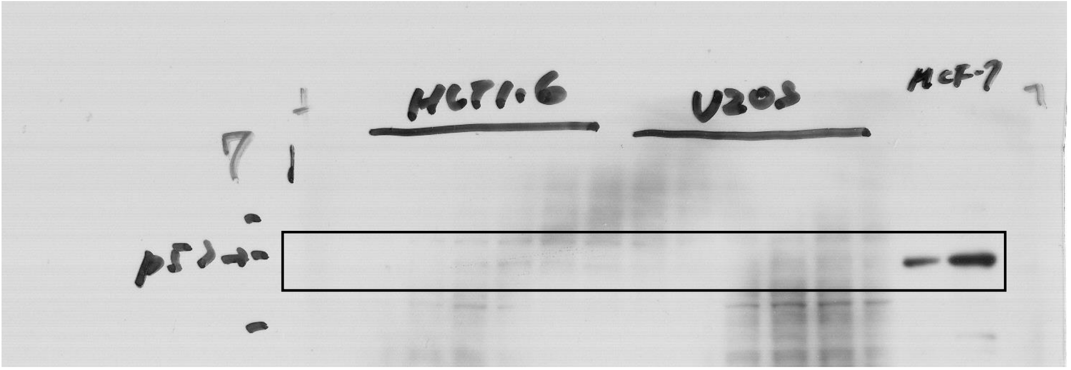

IB: β-actin (HCT116, U2OS)

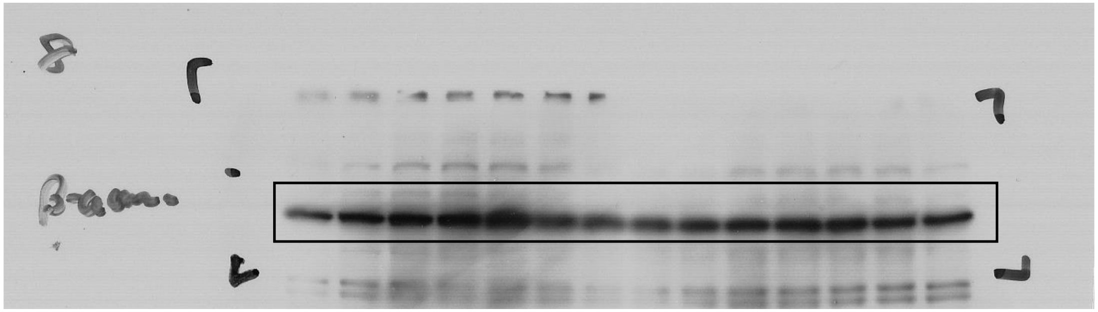

Fig. 6A, 6B **Fig. 10G**

IB: p-MEK **IB: MEK**

Fig. 6A

IB: p-MEK (Coffee)

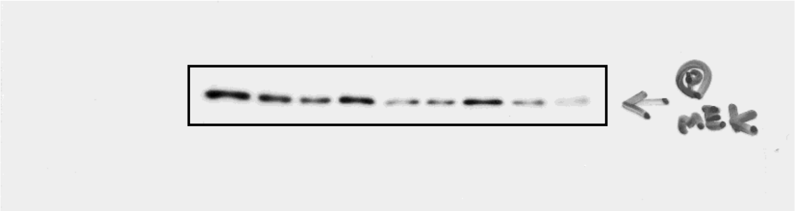

Fig. 6A

IB: MEK (Coffee)

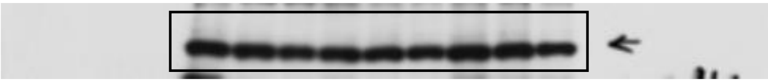

Fig. 6B

IB: p-MEK (Decaf)

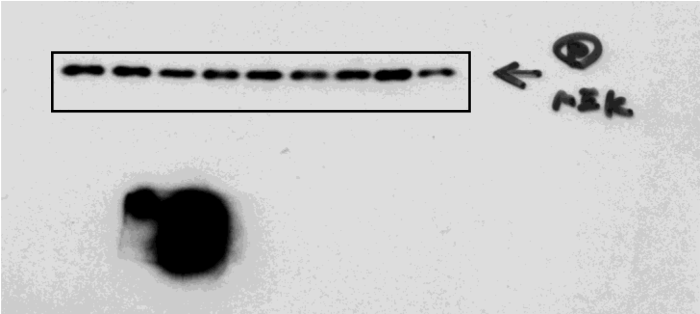

Fig. 6B

IB: MEK (Decaf)

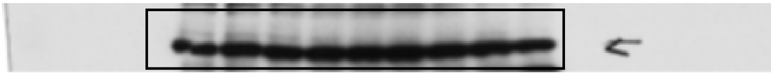

Fig. 10G

IB: p-MEK (Caffeine)

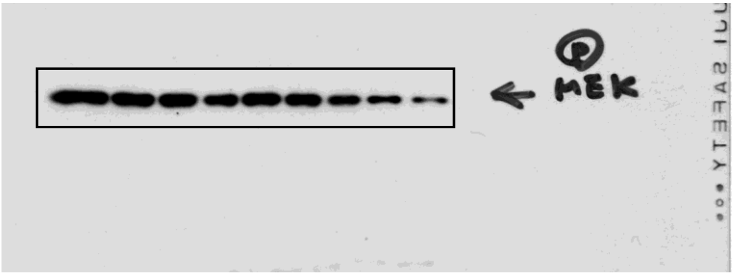

Fig. 10G

IB: MEK (Caffeine)

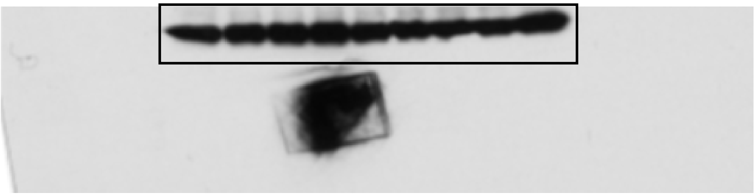

Fig. 6A, 6B **Fig. 10G**

IB: p-ERK

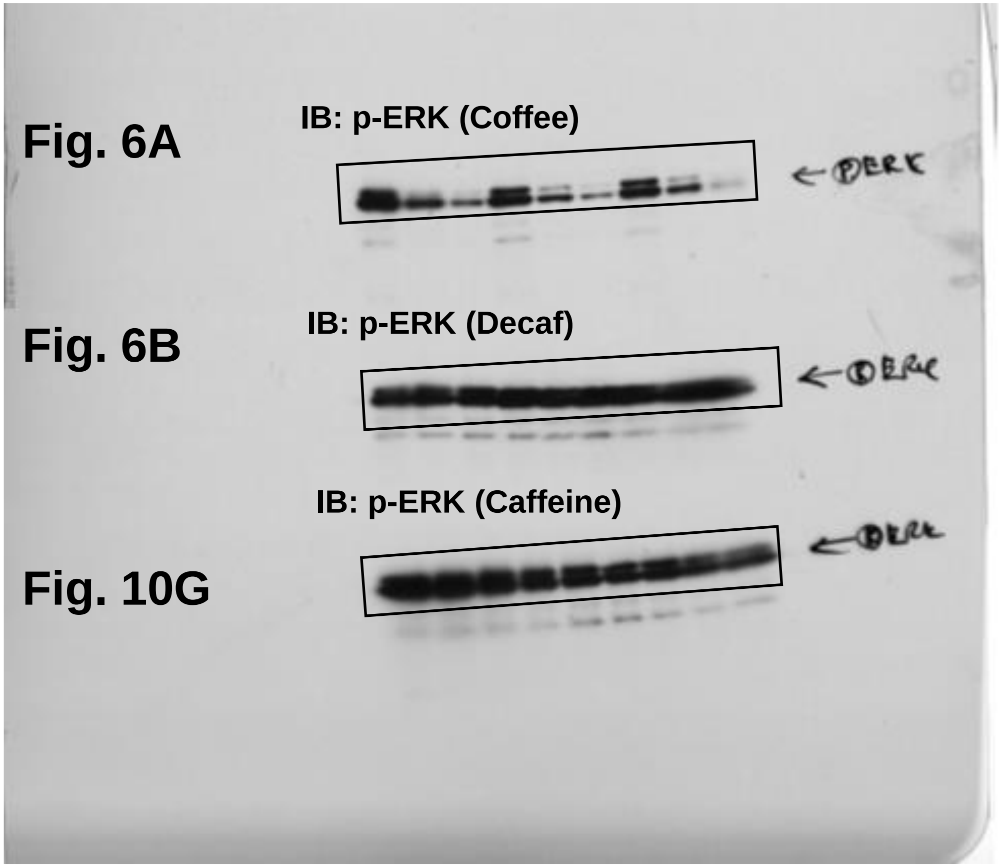

IB: ERK

Fig. 6A **Fig. 6B**

IB: ERK (Coffee) IB: ERK (Decaf)

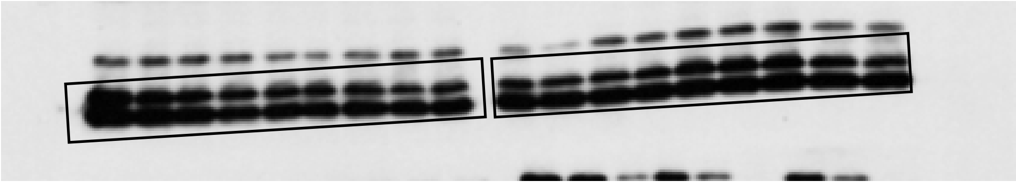

Fig. 10G

IB: ERK (Caffeine)

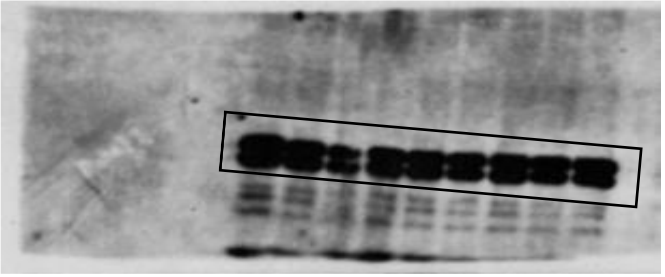

Fig. 6A, 6B

Fig. 10G

IB: p-Akt

Fig. 6A

IB: p-Akt (Coffee)

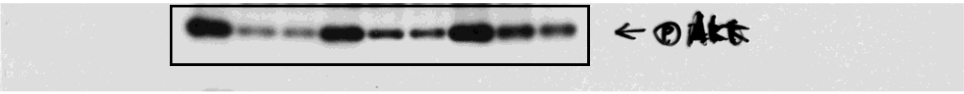

Fig. 6B

IB: p-Akt (Decaf)

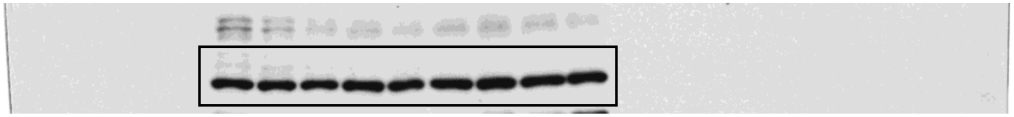

Fig. 10G

IB: p-Akt (Caffeine)

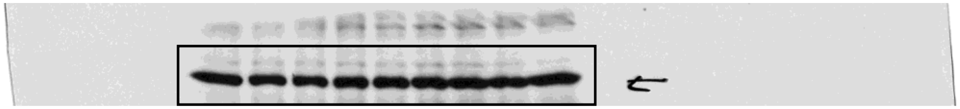

IB: Akt

Fig. 6A

IB: Akt (Coffee)

Fig. 6B

IB: Akt (Decaf)

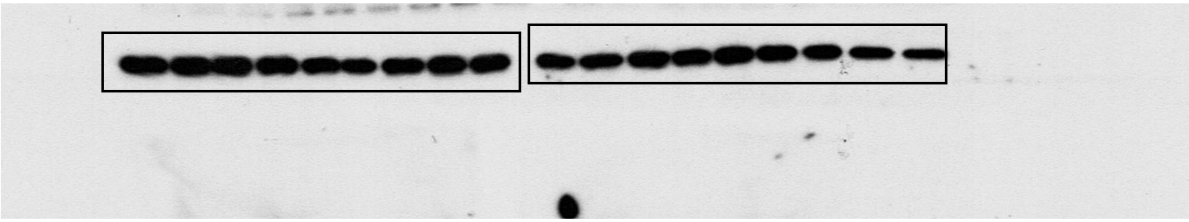

Fig. 10G

IB: Akt (Caffeine)

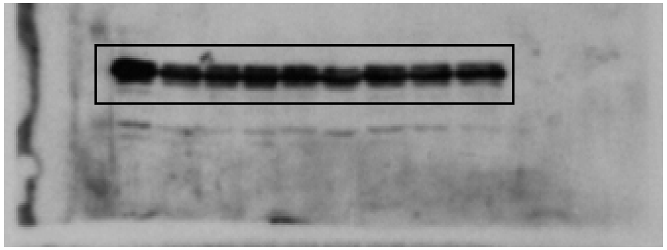

Fig. 7A

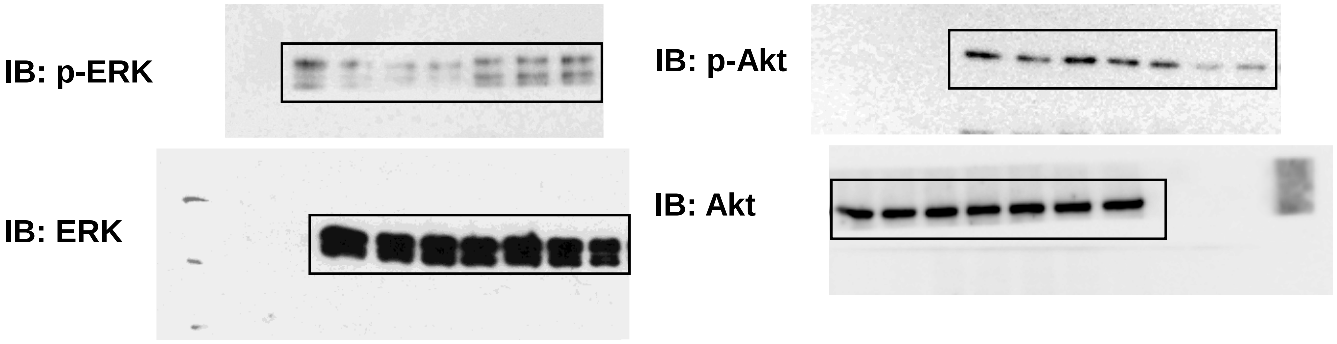
IB: p-ERK
IB: p-Akt
IB: ERK
IB: Akt

Fig. 7D

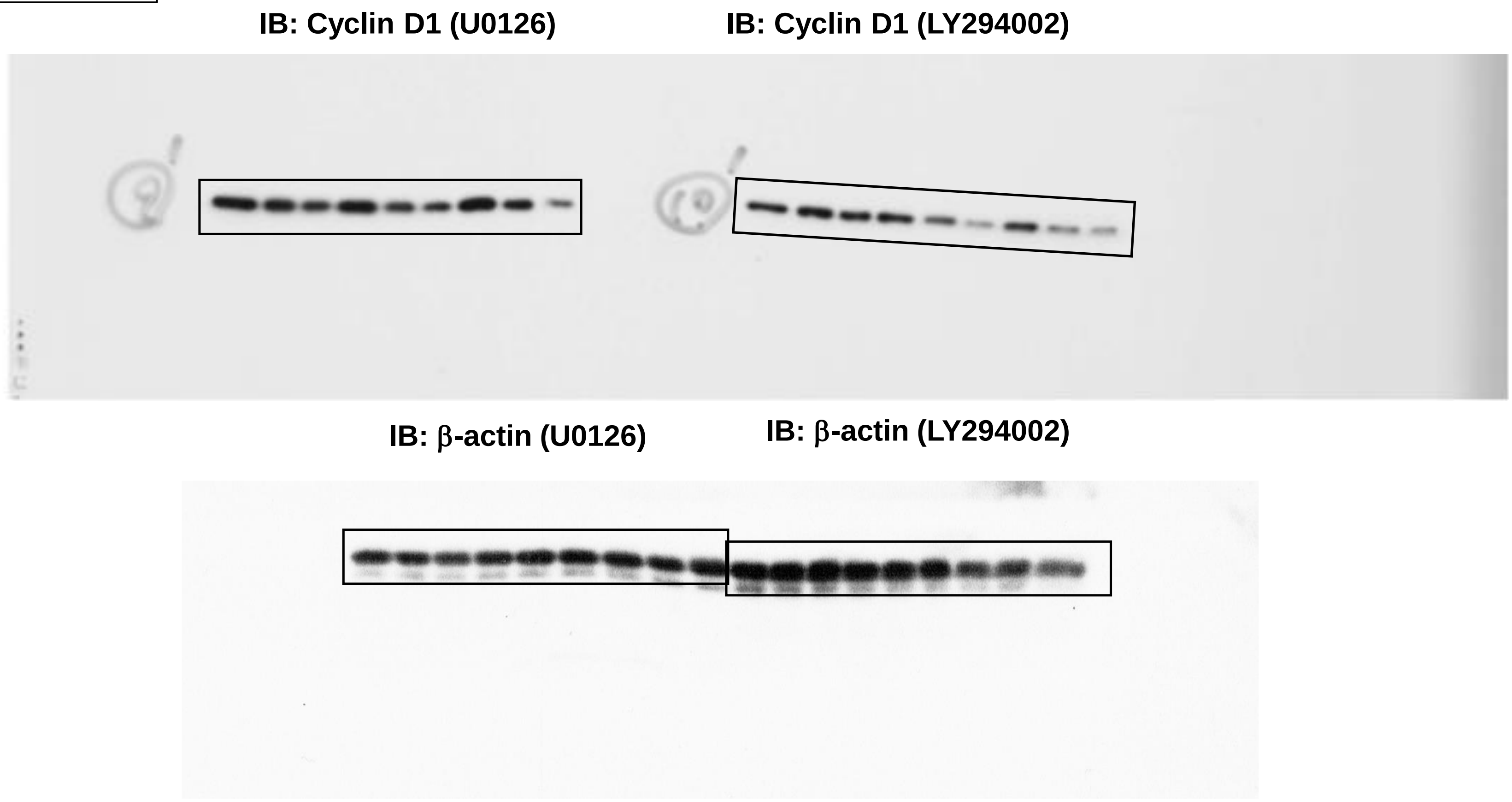
IB: Cyclin D1 (U0126)
IB: Cyclin D1 (LY294002)
IB: β-actin (U0126)
IB: β-actin (LY294002)

Fig.8A

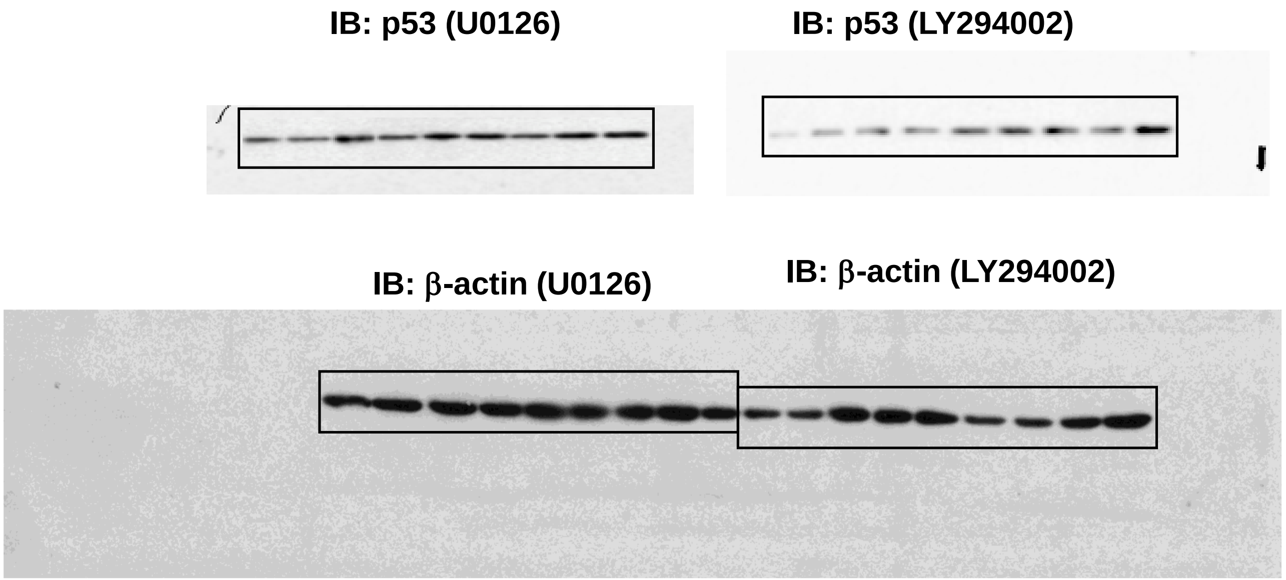
IB: p53 (U0126)
IB: p53 (LY294002)
IB: β-actin (U0126)
IB: β-actin (LY294002)

Fig. 9A

IB: ERα

Caffeine (ERα) **Caffeic acid (ERα)** **Pyrocatechol (ERα)** **Trigonelline (ERα)** **Chlorogenic acid (ERα)**

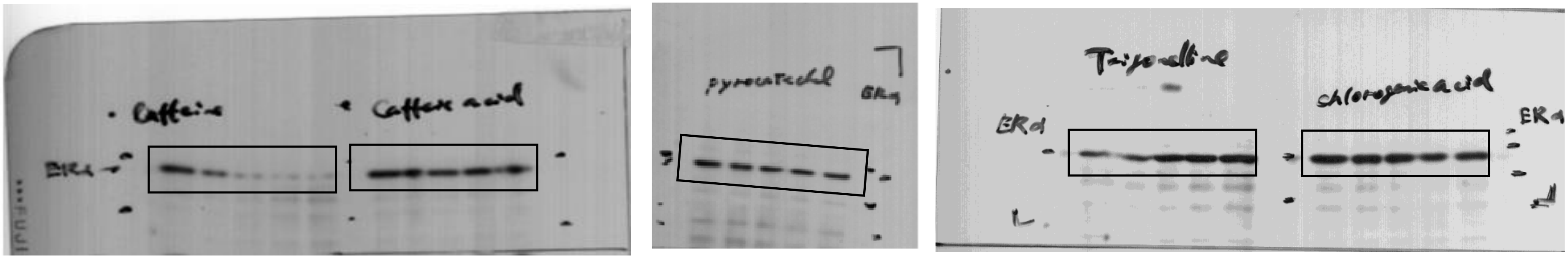

IB: β-actin

Caffeine (β-actin) **Caffeic acid (β-actin)** **Pyrocatechol (β-actin)**

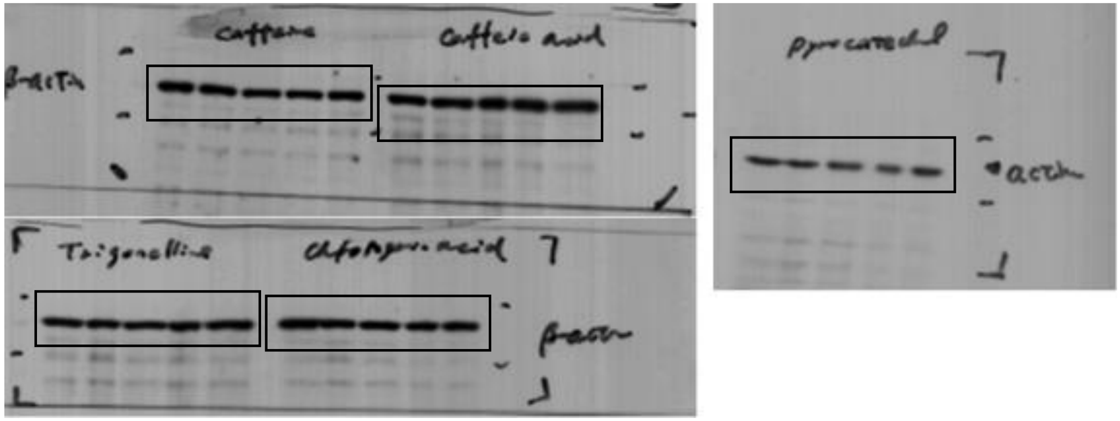

Trigonelline (β-actin) **Chlorogenic acid (β-actin)**

Fig. 10C

IB: Cyclin D1 (Caffeine)

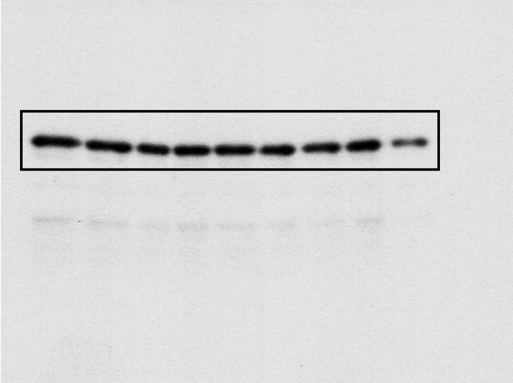

IB: β-actin (Caffeine)

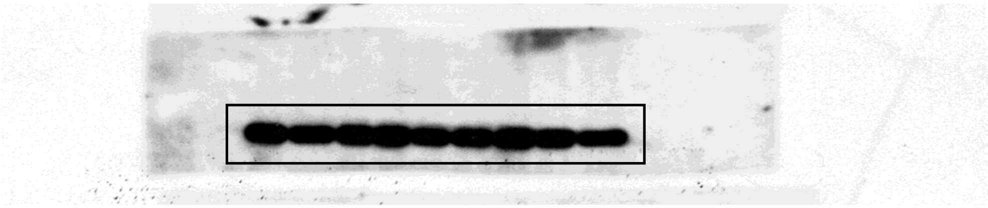

Fig. 10E

IB: p53 (Caffeine)

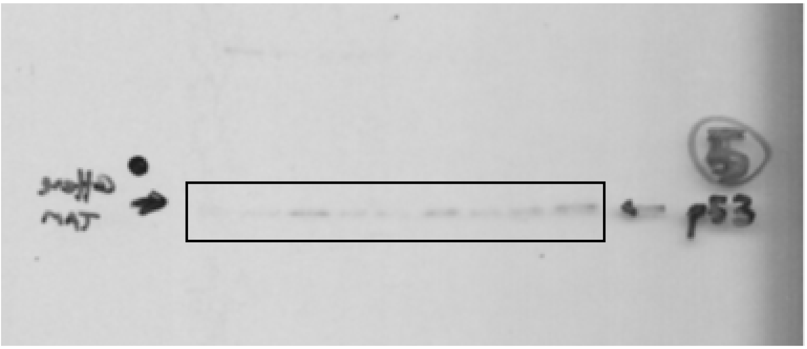

IB: β-actin (Caffeine)

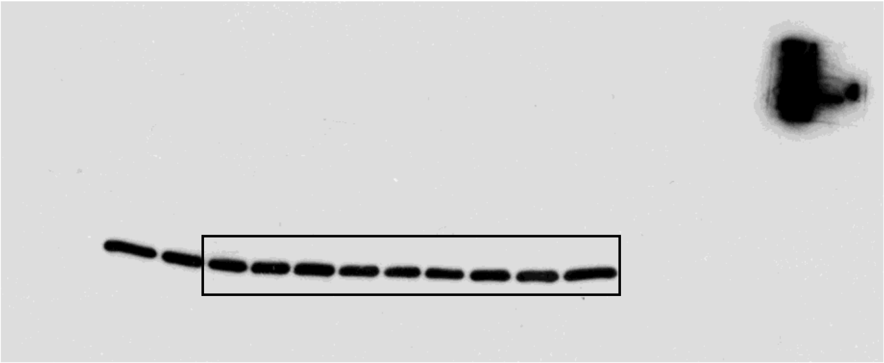

Fig.11C

IB: Cyclin D1

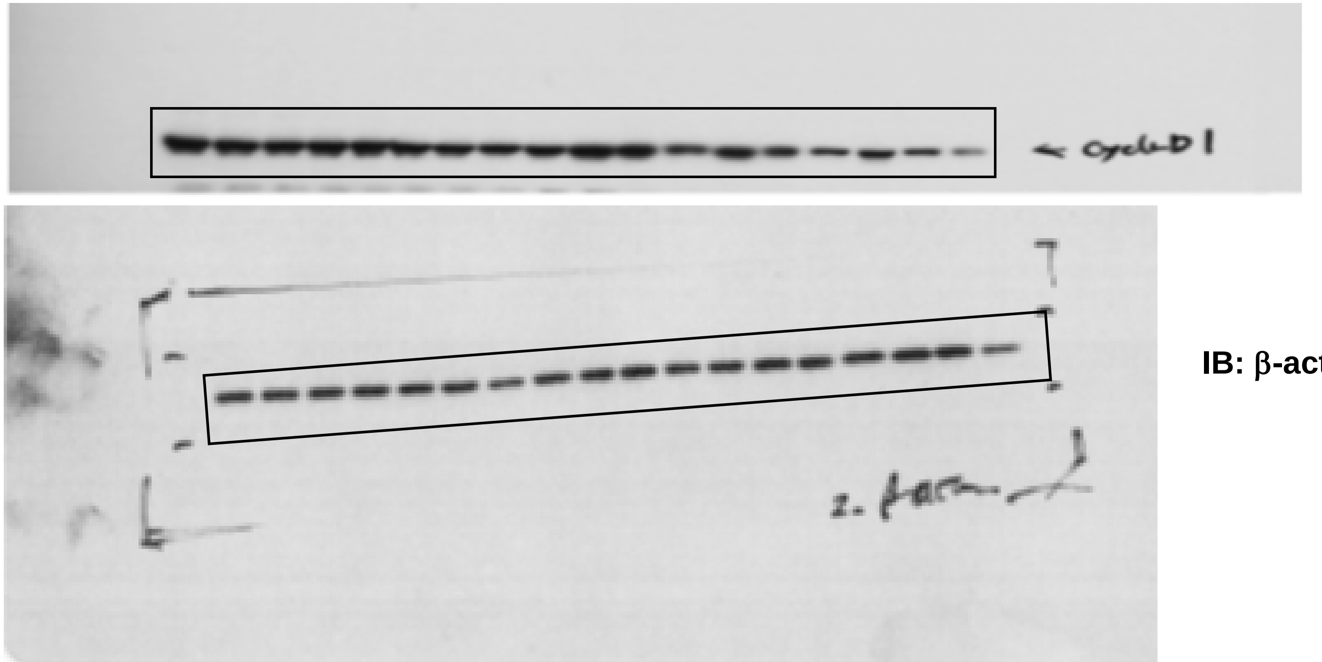

IB: β-actin

Fig.12A

IB: p53

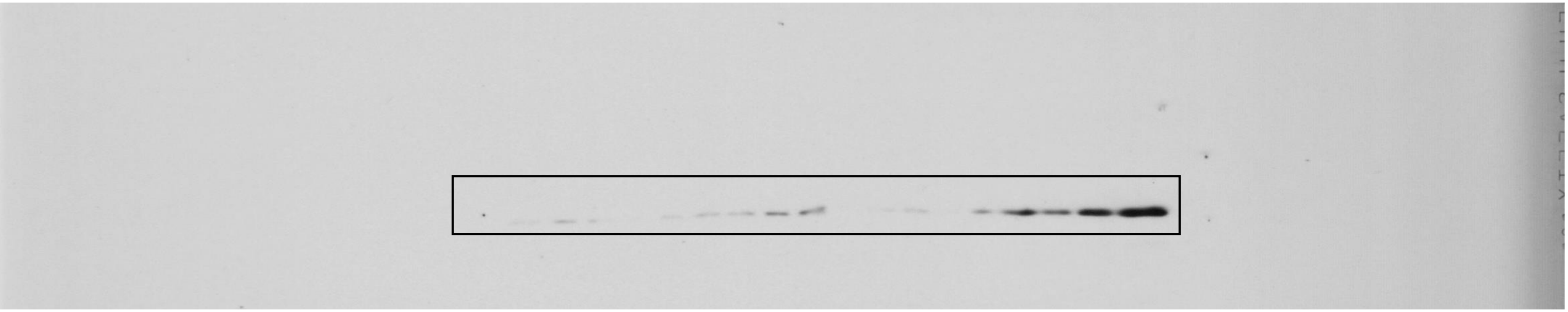

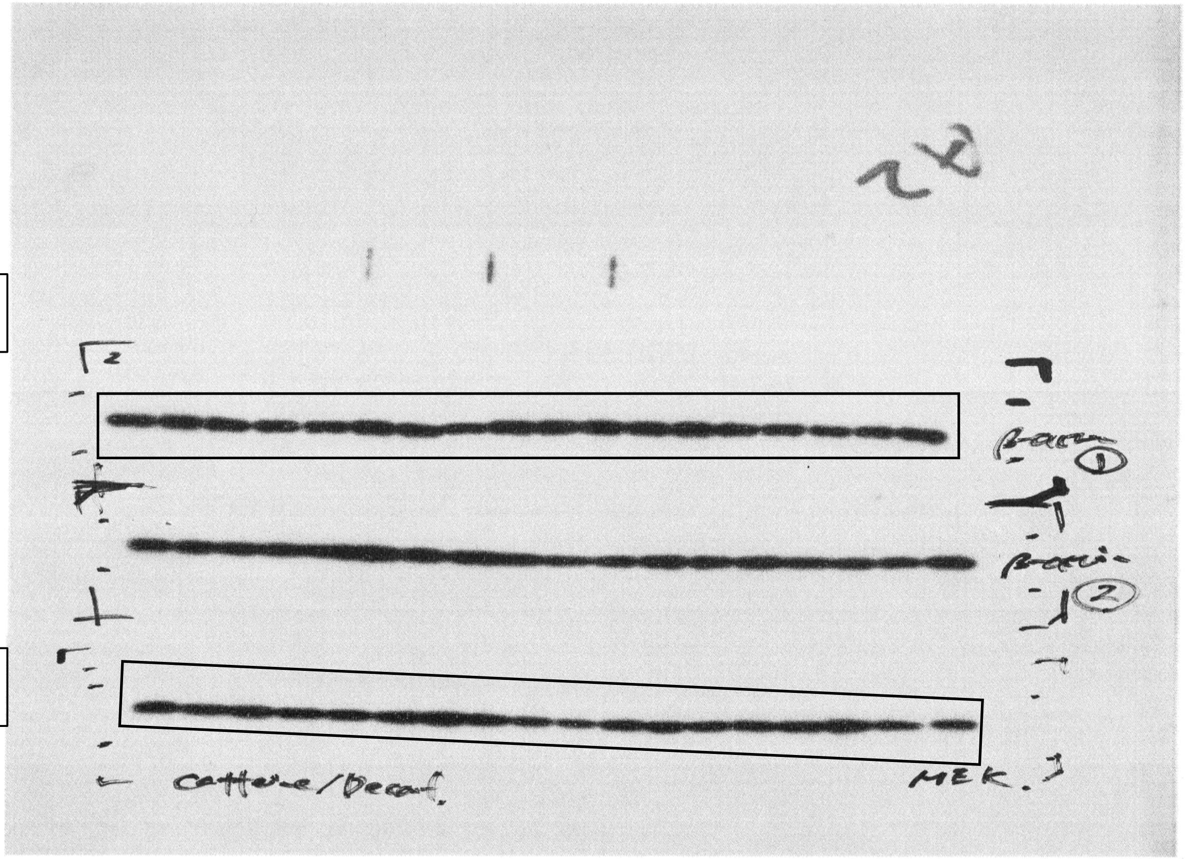

Fig.12A

IB: β-actin

Fig.12C

IB: MEK

Fig.12C

IB: p-MEK

IB: p-ERK

IB: ERK

IB: p-Akt

IB: Akt

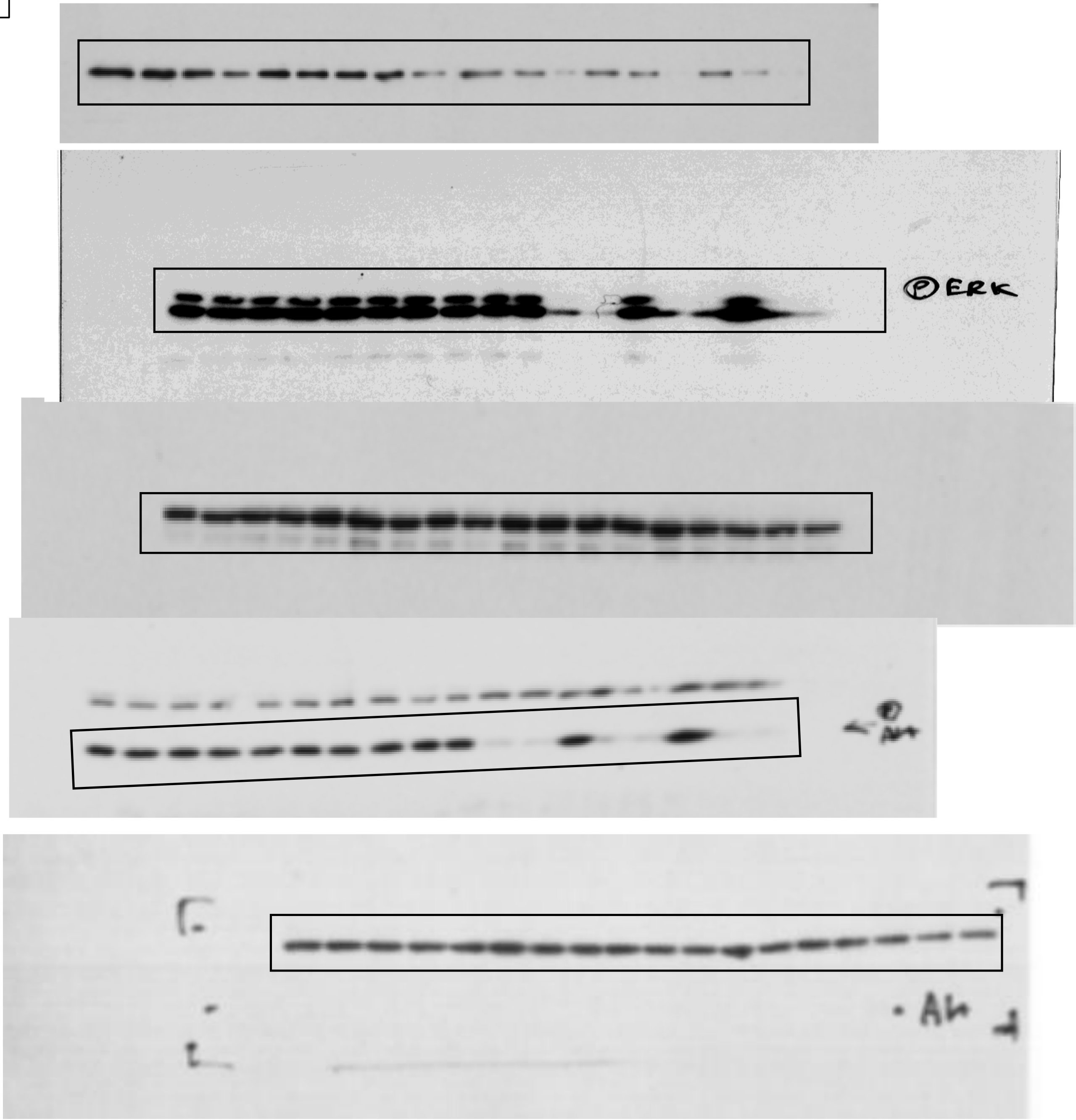

Supplemental Figure S2

IB: ERα

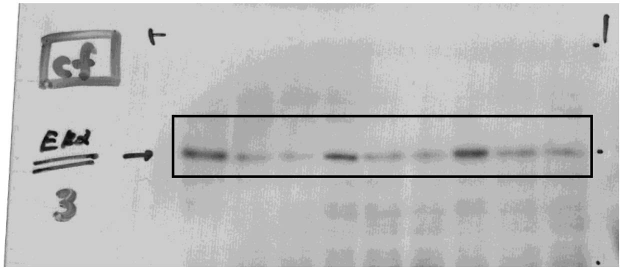

IB: p53

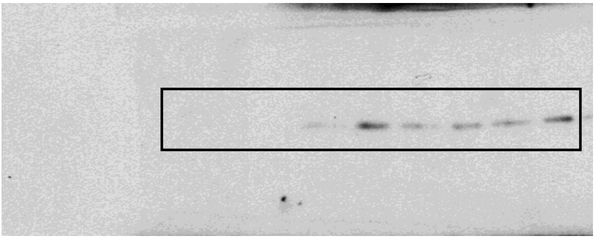

IB: β-actin

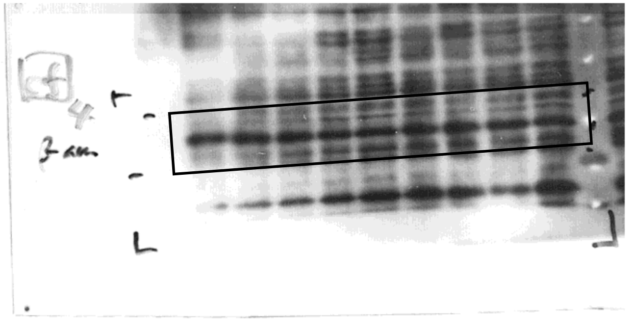

Supplement: Supplementary file 1 — Supplementary Figures. [file 41598_2020_76445_MOESM1_ESM.pdf]
